# A Multi-Component Model of Hodgkin's Lymphoma

**DOI:** 10.1371/journal.pone.0124614

**Published:** 2015-04-27

**Authors:** Martin S. Staege

**Affiliations:** Department of Pediatrics, Martin Luther University Halle-Wittenberg, Halle (S), Germany; University of Pittsburgh, UNITED STATES

## Abstract

Hodgkin’s lymphoma is an example for a tumor with an extremely tight interaction of tumor cells with cells from the tumor micro-environment. These so-called bystander cells are not inert but interact actively with the tumor cells. Some of these cells support tumor growth by delivery of co-stimulating and anti-apoptotic signals (“helper cells”). Other cells (“killer cells”) are involved in the anti-tumor immune response which is obviously not efficient enough for tumor elimination. The activity of both helper cells and killer cells is regulated by additional cells in the stroma (“regulatory cells”). The dynamic behavior of such multi-component systems is difficult to predict. In the present paper we propose a model that can be used for simulation of essential features of this system. In this model, tumor growth depends on (i) presence of few cancer stem cells, (ii) co-stimulation of cancer cells by the tumor stroma, (iii) activity of regulatory cells that suppress killer cells without suppression of helper cells. The success of cytotoxic/cytostatic therapy in this model varies depending on the therapy-related toxicity for each of the cell populations. The model also allows the analysis of immunotherapeutic interventions. Under certain conditions, paradox enhancement of tumor growth can occur after therapeutic intervention. The model might be useful for the design of new treatment strategies for Hodgkin’s lymphoma and other tumors with prominent tumor-stroma interaction.

## Introduction

Hodgkin’s lymphoma (HL) is a hematopoietic malignancy of uncertain origin. A key feature of HL is the presence of a very low number of tumor cells and a background of high numbers of non-malignant cells that are attracted by chemokines secreted by the tumor cells. The non-malignant component of the tumor is not passive but is actively involved in regulation of tumor growth [1]. HL cells undergo spontaneous apoptosis [2] which might be one of the reasons why the number of successfully established HL cell lines is very low [3]. *In vivo*, anti-apoptotic signals from the stroma counteract spontaneous apoptosis and allow tumor growth.

The non-malignant component in HL is a mixture of different cell types including T helper cells, cytotoxic T cells, regulatory T cells, macrophages, and other cell types. Some of these cell types support tumor growth by delivery of growth-stimulating and anti-apoptotic signals [1]. Other cell types are involved in the immune response against the tumor. In growing tumors, this immune response is obviously not able to control the tumor. However, HL cells can be attacked by cytotoxic cells, and adoptive transfer of antigen-specific T cells has been used for treatment of patients with HL [4,5].

Growth of HL depends on the equilibrium between pro- and anti-tumoral activities of the tumor micro-environment. These activities are regulated in part by regulatory T cells. However, the impact of regulatory T cells on survival of HL patients is unclear. Some studies suggest that high numbers of regulatory T cells are a favorable prognostic marker [6–8]. Other studies suggest that regulatory T cells can inhibit the anti-tumor activity of T cells [9] or have no impact on survival [10]. Heterogeneity of the regulatory T cell population or individual differences in the equilibrium between anti- and pro-tumoral effects of regulatory T cells might account for these observations. Surprisingly, high numbers of cytotoxic T cells seem to be an unfavorable prognostic marker [6–8,11] which might be indicative for insufficient activity of these cells.

Like in other tumors, the tumor cell population in HL is heterogeneous. In addition to a population of extraordinary, large, often multinucleated cells (Hodgkin/Reed-Sternberg cells) that can be considered as the mature tumor cells (MTC), a population of cancer stem cells (CSC) seems to be present [12–14].

The dynamic behavior of multi-component systems like HL is difficult to predict. Therefore, we developed an *in-silico* model that can be used for the simulation of essential features of this tumor.

## Material and Model

**Fig 1:**
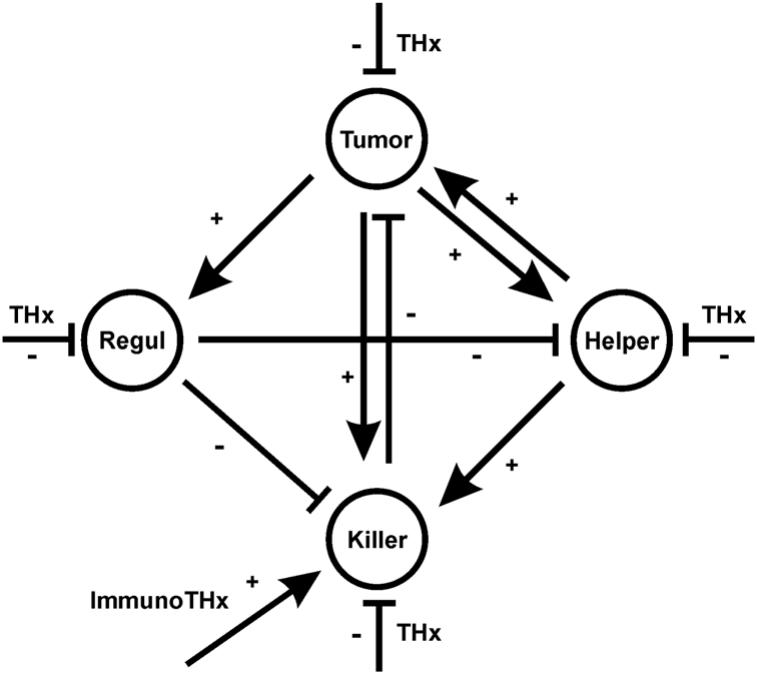
Cellular interactions in the Hodgkin’s lymphoma model. The model consists of 4 cell compartments (the tumor cells (Tumor), “Helper Cells” (Helper), “Killer Cells” (Killer) and “Regulatory Cells” (Regul). All compartments can be targeted by cytotoxic therapy (THx). In addition, Immunotherapy (ImmunoTHx) can increase number and activity of killer cells. Tumor cells can stimulate (+) all other cell types. “Helper Cells” increase the viability of tumor cells and supply help for “Killer Cells.” “Regulatory Cells” suppress (-) “Killer Cells” as well as “Helper Cells”. Finally, “Killer Cells” can kill tumor cells.

The basic structure of the model is depicted in Fig 1. The model consists of 4 compartments: the tumor cell compartment, “Killer Cells”, “Helper Cells”, and “Regulatory Cells”. The *in-vivo* equivalents of “Killer Cells”, “Helper Cells” and “Regulatory Cells” can be different T cell types (cytotoxic T cells, T helper cells, regulatory T cells) but are not necessarily restricted to T cells. For example, “Killer Cells” can also include natural killer (NK) cells and “Helper Cells” can include macrophages. For simplicity, these compartments are not further subdivided in the model.

**Fig 2:**
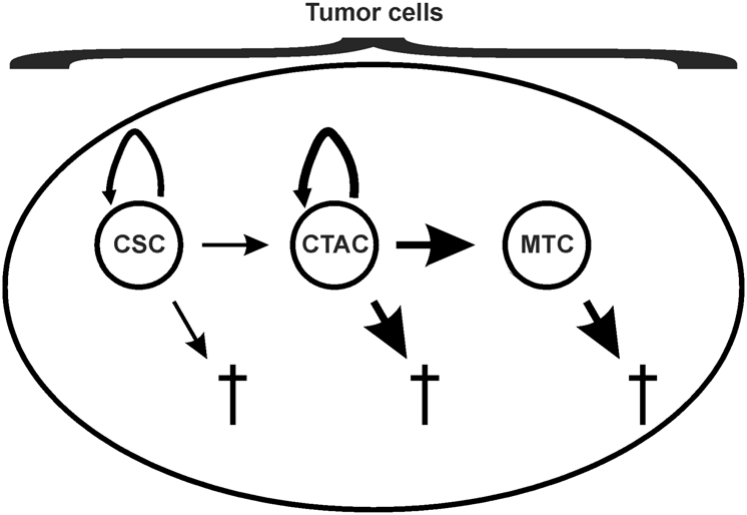
The tumor cell compartments of the model. The model assumes three types of tumor cells. In addition to mature tumor cells (MTC), cancer stem cells (CSC), and cancer transit amplifying cells (CTAC) are assumed. CTAC represent the major proliferating population of tumor cells with high differentiation capacity. Without replenishment from the CSC pool, this population will completely differentiate into MTC. CSC are considered as small population with low proliferative activity but high self-renewal capacity. All cell populations are continuously lost by natural cell death which is more pronounced in MTC and CTAC than in CSC.

We divided the tumor cell compartment into three sub-compartments (Fig 2). In addition to mature tumor cells (MTC) without proliferation capacity, we included a slow dividing cancer stem cell compartment (CSC) and a fast dividing transit amplifying cell compartment (CTAC). The principle behavior of the model does not depend on the presence of multiple separate tumor cell compartments. If desired, it is possible to use only one compartment as a universal tumor cell compartment.

**Fig 3:**
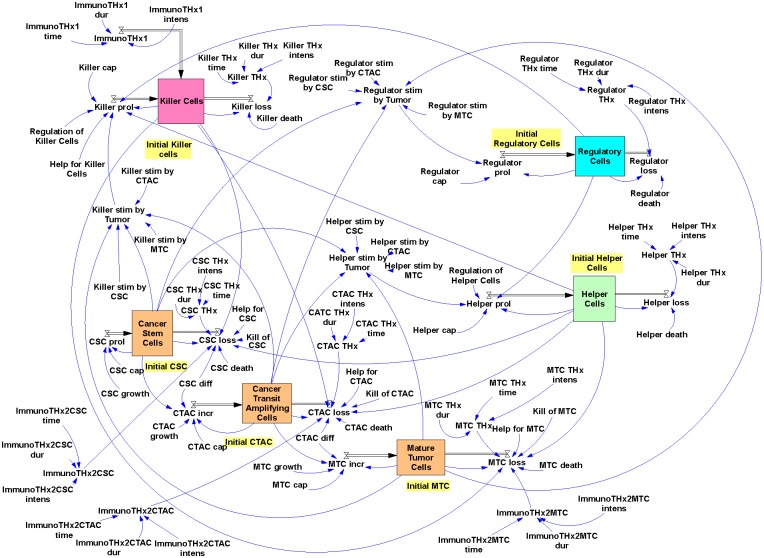
The Hodgkin’s lymphoma model. A snapshot from the model programmed in Vensim is presented. Details are explained in the text.

The complete model (Fig 3) was created with Vensim PLE for Windows Version 5.9e (Ventana Systems, Salisbury, Wiltshire, UK).

The model has the following features: Proliferation of CSC (CSC prol) depends on the CSC growth rate (CSC growth) and is limited by a CSC specific capacity constant (CSC cap). This capacity constant and the corresponding capacity constants in the other cell compartments (CTAC cap, MTC cap, Killer cap, Helper cap, and Regulator cap) are used for simulation of resource limits. CSC are continuously lost by (i) natural death, depending on a CSC specific death rate (CSC death), (ii) killing of CSC by cells from the “Killer Cell” compartment (Kill of CSC), and (iii) differentiation of CSC into cells that have lost the stem cell features (CSC diff). Loss of CSC is inhibited by anti-apoptotic signals from the “Helper Cell” compartment (Help for CSC).

Increase of CTAC (CTAC incr) is dependent on the differentiation of cells from the CSC compartment (CSC diff). In addition, CTAC proliferate with a CTAC specific proliferation rate (CTAC growth) and are limited by CTAC cap. CTAC are continuously lost by (i) natural death, depending on the CTAC specific death rate (CTAC death), (ii) killing of CATC by cells from the “Killer Cell” compartment (Kill of CTAC), and (iii) differentiation into mature tumor cells (CTAC diff). Loss of CTAC is inhibited by anti-apoptotic signals from the “Helper Cell” compartment (Help for CTAC).

Increase of MTC (MTC incr) depends on the differentiation of cells from the CTAC compartment (CTAC diff). In principle, these cells can proliferate with a MTC specific proliferation rate (MTC growth). In the default setting, we set this growth rate to zero. MTC incr is limited by MTC cap, and MTC are continuously lost by natural death that is dependent on a MTC specific death rate (MTC death), and killing of MTC by cells from the “Killer Cell” compartment (Kill of MTC). MTC loss is inhibited by anti-apoptotic signals from the “Helper Cell” compartment (Help for MTC). For MTC as well as CTAC, proliferation (prol) was replaced by increase (incr) because in these two sub-compartments cells are newly formed not only by proliferation but also by differentiation from progenitor cell compartments.

Proliferation of “Helper Cells” (Helper prol) is dependent on the stimulation by tumor cells. For each tumor cell compartment (CSC, CTAC, MTC) a “Helper Cell”-specific stimulation factor (Helper stim by CSC, Helper stim by CTAC, Helper stim by MTC) is used. “Helper Cell” proliferation is limited by Helper cap and suppressed by regulation from cells in the “Regulatory Cell” compartment (Regulation of Helper Cells). “Helper Cells” are continuously lost by natural death that depends on a specific death rate (Helper death).

Similarly, proliferation of “Killer Cells” (Killer prol) is dependent on the stimulation by tumor cells. Again, for each tumor cell compartment a “Killer Cell”-specific stimulation factor is used. Killer cell proliferation is limited by Killer cap, suppressed by regulation from cells in the “Regulatory Cell” compartment (Regulation of Killer Cells) and supported by helper activity from cells in the “Helper Cell” compartment (Help for Killer Cells). “Killer Cells” are continuously lost by natural death that depends on a specific death rate (Killer death).

Finally, proliferation of “Regulatory Cells” (Regulator prol) is, again, dependent on the stimulation by tumor cells. For each tumor cell compartment a “Regulatory Cell”-specific stimulation factor is used. Proliferation of “Regulatory Cells” is limited by Regulator cap. “Regulatory Cells” are continuously lost by natural death which is dependent on a specific death rate.

For simulation of therapeutic interventions, therapy factors were included in the model. These factors increase the cell loss in the different compartments. For maximal flexibility, therapy intensity (THx intens), starting time of the therapy (THx time) and duration of the therapy (THx dur) can be set independently for all cell compartments. These parameters can be adjusted in order to simulate different toxicity and pharmacokinetics for the different cell compartments.

Immunotherapy extensions of the model are described below.

The default values of all parameters are summarized in Table 1. Equations used for calculations in the model are summarized in Table 2. If not otherwise stated, the model was simulated with the following settings: initial time: 0; final time: 2000 or 4000; time step: 0.007812; save results at SAVEPER = 1; units for time: day; integration type: Euler.

**Table 1:** Default values used in the model (see Fig 3).

Parameter	Value
CSC cap	1e-009
CSC death	0.01
CSC diff	0.002
CSC growth	0.01
CSC Thx dur	100
CSC THx intens	0
CSC THx time	400
CTAC cap	1e-009
CTAC death	0.03
CTAC diff	0.91
CTAC growth	0.9
CTAC Thx dur	100
CTAC THx intens	0
CTAC THx time	400
Help for CSC	0.001
Help for CTAC	0.001
Help for Killer Cells	1e-005
Help for MTC	0.001
Helper cap	1e-009
Helper death	1e-005
Helper stim by CSC	0.001
Helper stim by CTAC	0.001
Helper stim by MTC	0.001
Helper Thx dur	100
Helper THx intens	0
Helper THx time	400
ImmunoTHx1 dur	1
ImmunoTHx1 intens	0
ImmunoTHx1 time	300
ImmunoTHx2CSC dur	3000
ImmunoTHx2CSC intens	0
ImmunoTHx2CSC time	1000
ImmunoTHx2CTAC dur	3000
ImmunoTHx2CTAC intens	0
ImmunoTHx2CTAC time	1000
ImmunoTHx2MTC dur	3000
ImmunoTHx2MTC intens	0
ImmunoTHx2MTC time	1000
Initial CSC	10
Initial CTAC	10
Initial Helper Cells	10
Initial Killer Cells	10
Initial MTC	10
Initial Regulatory Cells	10
Killer cap	1e-009
Killer death	1e-005
Killer stim by CSC	0.0001
Killer stim by CTAC	0.0001
Killer stim by MTC	0.02
Killer THx dur	100
Killer THx intens	0
Killer THx time	400
Kill of CSC	1e-006
Kill of CTAC	1e-006
Kill of MTC	1e-006
MTC cap	1e-009
MTC death	0.5
MTC growth	0
MTC Thx dur	100
MTC THx intens	0
MTC THx time	400
Regulation of Helper Cells	0.0001
Regulation of Killer Cells	0.5
Regulator cap	0.0001
Regulator death	1e-005
Regulator stim by CSC	0.001
Regulator stim by CTAC	0.001
Regulator stim by MTC	0.0001
Regulator Thx dur	100
Regulator THx intens	0
Regulator THx time	400

Default values allow the simulation of essential features of Hodgkin’s lymphoma. Other tumor types might require adjustments.

**Table 2:** Equations used in the model (see Fig 3).

Variable	Equation
Cancer Stem Cells	INTEG (IF THEN ELSE (Cancer Stem Cells + CSC prol—CSC loss > 0, + CSC prol—CSC loss,—Cancer Stem Cells), Initial CSC)
Cancer Transit Amplifying Cells	INTEG (IF THEN ELSE ((Cancer Transit Amplifying Cells + CTAC incr—CTAC loss) > 0, + CTAC incr—CTAC loss, -Cancer Transit Amplifying Cells), Initial CTAC)
CSC loss	(MAX (0, (Cancer Stem Cells * CSC death) + ((1 + ImmunoTHx2CSC) * Kill of CSC * Killer Cells)—(Helper Cells * Help for CSC)) + (Cancer Stem Cells * CSC diff)) + (Cancer Stem Cells * CSC THx)
CSC prol	MAX (0, (CSC growth * Cancer Stem Cells)—(Cancer Stem Cells * Cancer Stem Cells * CSC cap))
CSC THx	PULSE (CSC THx time, CSC THx dur) * CSC THx intens
CTAC incr	MAX (0, (CSC diff * Cancer Stem Cells) + (CTAC growth * Cancer Transit Amplifying Cells)—(CTAC cap * Cancer Transit Amplifying Cells *Cancer Transit Amplifying Cells))
CTAC loss	(MAX (0, (Killer Cells * Kill of CTAC * (1 + ImmunoTHx2CTAC)) + (CTAC death * Cancer Transit Amplifying Cells)—(Helper Cells * Help for CTAC)) + (CTAC diff * Cancer Transit Amplifying Cells)) + (CTAC THx * Cancer Transit Amplifying Cells)
CTAC THx	PULSE (CTAC THx time, CTAC THx dur) * CTAC THx intens
Helper Cells	INTEG (IF THEN ELSE ((Helper Cells + Helper prol—Helper loss) > 0, (+Helper prol—Helper loss),—Helper Cells), Initial Helper Cells)
Helper loss	(Helper Cells * Helper death) + (Helper Cells * Helper THx)
Helper prol	IF THEN ELSE (Helper Cells * Helper stim by Tumor > 0, (MAX (0, (Helper stim by Tumor * Helper Cells)-(Helper Cells * Helper Cells * Helper cap)—(Regulation of Helper Cells * Regulatory Cells))), 0)
Helper stim by Tumor	(Cancer Stem Cells * Helper stim by CSC) + (Helper stim by CTAC * Cancer Transit Amplifying Cells) + (Mature Tumor Cells * Helper stim by MTC)
Helper Thx	PULSE (Helper THx time, Helper THx dur) * Helper THx intens
ImmunoTHx1	PULSE (ImmunoTHx1 time, ImmunoTHx1 dur) * ImmunoTHx1 intens
ImmunoTHx2CSC	PULSE (ImmunoTHx2CSC time, ImmunoTHx2CSC dur) * ImmunoTHx2CSC intens
ImmunoTHx2CTAC	PULSE (ImmunoTHx2CTAC time, ImmunoTHx2CTAC dur) * ImmunoTHx2CTAC intens
ImmunoTHx2MTC	PULSE (ImmunoTHx2MTC time, ImmunoTHx2MTC dur) * ImmunoTHx2MTC intens
Killer Cells	INTEG (IF THEN ELSE ((Killer Cells + Killer prol—Killer loss + ImmunoTHx1) > 0, (+ Killer prol—Killer loss + ImmunoTHx1),—Killer Cells), Initial Killer Cells)
Killer loss	(Killer Cells * Killer death) + (Killer THx * Killer Cells)
Killer prol	IF THEN ELSE (Killer Cells * Killer stim by Tumor * Help for Killer Cells * Helper Cells > 0, (MAX (0, (Killer Cells * Killer stim by Tumor)—(Regulation of Killer Cells * Regulatory Cells) + (Helper Cells * Help for Killer Cells)—(Killer Cells * Killer Cells * Killer cap))), 0)
Killer stim by Tumor	MAX (0, ((Cancer Stem Cells * Killer stim by CSC) + (Killer stim by CTAC * Cancer Transit Amplifying Cells) + (Mature Tumor Cells * Killer stim by MTC)))
Killer THx	PULSE (Killer THx time, Killer THx dur) * Killer THx intens
Mature Cancer Cells	INTEG (IF THEN ELSE ((Mature Tumor Cells + MTC incr—MTC loss) > 0, + MTC incr—MTC loss,—Mature Tumor Cells), Initial MTC)
MTC incr	MAX (0, (MTC growth * Mature Tumor Cells) + (CTAC diff * Cancer Transit Amplifying Cells)—(MTC cap * Mature Tumor Cells * Mature Tumor Cells))
MTC loss	(MAX (0, (Killer Cells * Kill of MTC * (1+ImmunoTHx2MTC)) + (MTC death * Mature Tumor Cells)—(Helper Cells * Help for MTC))) + (MTC THx * Mature Tumor Cells)
MTC THx	PULSE (MTC THx time, MTC THx dur) * MTC THx intens
Regulator loss	(Regulatory Cells * Regulator death) + (Regulator THx * Regulatory Cells)
Regulator prol	IF THEN ELSE (Regulatory Cells * Regulator stim by Tumor > 0, (MAX (0, (Regulatory Cells * Regulator stim by Tumor)—(Regulatory Cells * Regulatory Cells * Regulator cap))), 0)
Regulator stim by Tumor	(Cancer Stem Cells * Regulator stim by CSC) + (Cancer Transit Amplifying Cells * Regulator stim by CTAC) + (Mature Tumor Cells * Regulator stim by MTC
Regulator THx	PULSE (Regulator THx time, Regulator THx dur) * Regulator THx intens
Regulatory Cells	INTEG (IF THEN ELSE ((Regulatory Cells + Regulator prol-Regulator loss) > 0, (+ Regulator prol—Regulator loss),—Regulatory Cells), Initial Regulatory Cells)

The syntax of the equations is in accordance to Vensim PLE for Windows Version 5.9e (Ventana Systems, Salisbury, Wiltshire, UK). The complete packaged Vensim model is available as S1 Model. Simulations of this model can be run with Vensim Model Reader (http://vensim.com/vensim-model-reader/).

## Results and Discussion

### Tumor growth without therapy

**Fig 4:**
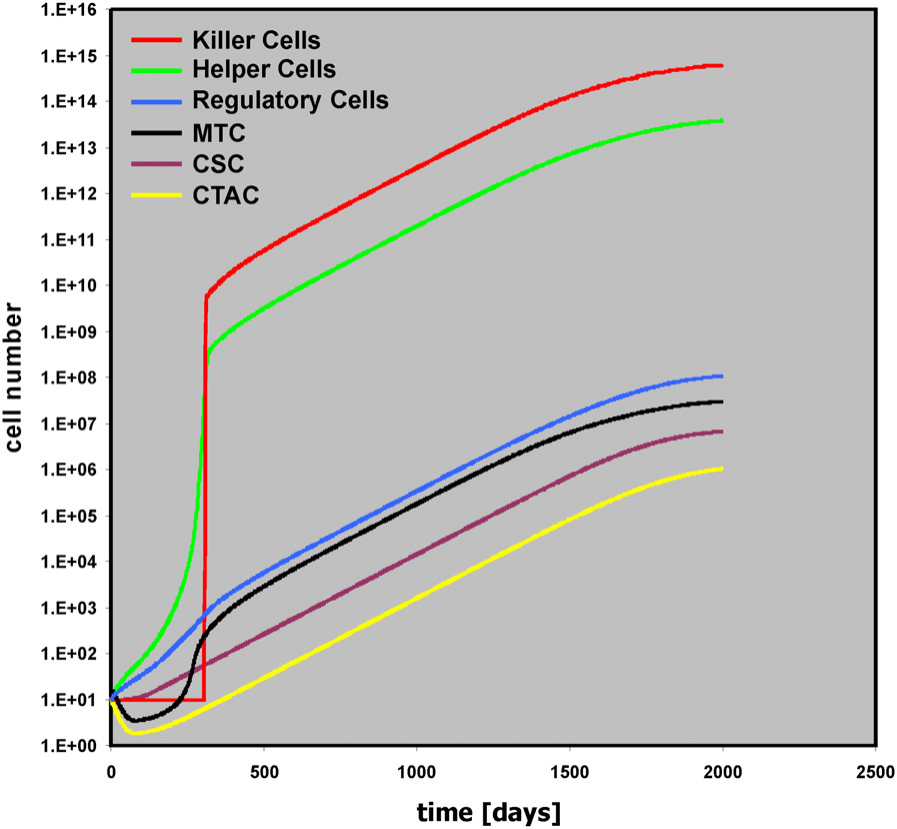
Tumor dynamics with default values of the model. Presented are time courses for all 6 cell populations with default values (see Table 1) of the model. With these default parameters massive tumor growth occurs.

The model in Fig 3 can simulate some of the essential features of Hodgkin’s lymphoma (HL). Using the default values, tumor growth occurs and the tumor mass consists mainly of “bystander cells” (Fig 4). Nevertheless, the driving force of the tumor growth is the proliferation of the tumor cell, especially in the CSC compartment. Without cancer stem cells (Initial CSC = 0), the numbers of other cell types in the model do not increase either and no tumor growth occurs (S1 Fig). Without cancer stem cell proliferation (CSC growth = 0), only small tumors will be obtained after a longer lag phase (S2 Fig). These tumors are a consequence of ongoing proliferation and differentiation of cells in the CTAC compartment leading to production of new MTC over a long time period. “Helper Cells” stimulated by these MTC inhibit complete loss of MTC. Such situations might occur if after tumor initiation the pool of cancer initiating stem cells is exhausted. In these cases, a steady state between cell death and death inhibition can result in a stable equilibrium. If in addition to the CSC sub-compartment the transit amplifying cell compartment also stops proliferation (CTAC growth = 0), tumor growth is completely absent (S3 Fig). A slight increase in the activity of “Killer Cells” against MTC (Kill of MTC = 5e-006) can also completely eliminate remaining MTC (S3 Fig).

In HL, tumor cell survival is dependent on the presence of anti-apoptotic signals from the micro-environment. The presented model shows the same behavior: Tumor growth depends on the presence of anti-apoptotic signals from a “Helper Cell” compartment. Without “Helper Cells” (Initial Helper Cells = 0) no tumor growth occurs (S4 Fig). In general, tumor growth can occur if CSC prol > CSC loss. Without “Helper Cells” and “Killer Cells” this situation occurs if CSC growth > (CSC death + CSC diff). In the presence of bystander cells, the amount of CSC loss is decreased (by “Helper Cells”) or increased (by “Killer Cells”). The final outcome depends on the relative strength of these two modifiers.

**Fig 5:**
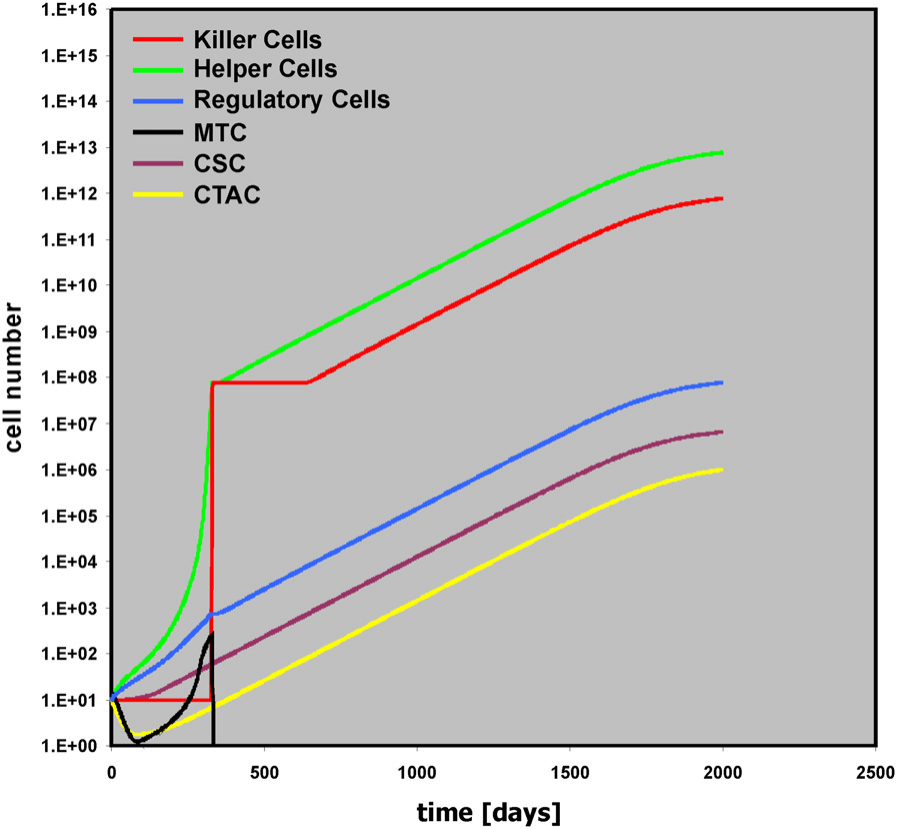
Insufficient tumor control despite increased cellular cytotoxicity against mature tumor cells. In this simulation the cytotoxicity of “Killer Cells” against mature tumor cells was increased (Kill of MTC = 0.1). Without killing of CSC, tumor control is insufficient.

With the default settings in Fig 4 the anti-tumor immune response is obviously not able to control tumor growth. Increasing the cytotoxic activity of the cells in the “Killer Cell” compartment can change this situation. If the cytotoxicity against the cancer stem cell compartment is high enough (Kill of CSC = 0.1), tumor growth is inhibited (S5 Fig). On the other hand, increasing only the cytotoxicity against the mature tumor cells is not sufficient to control tumor growth completely (Fig 5).

**Fig 6:**
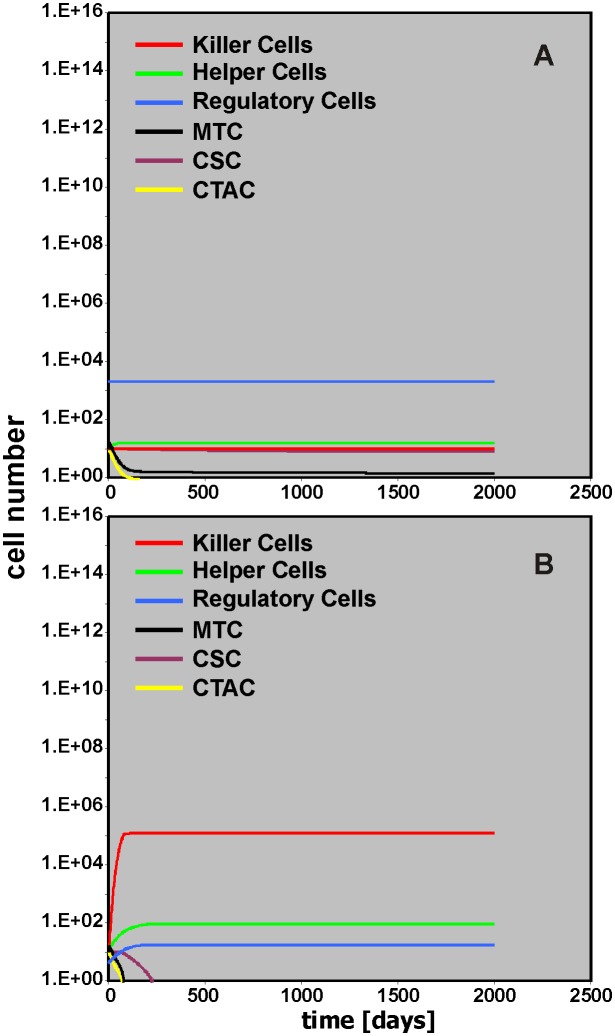
Impact of “Regulatory Cells” on tumor control. In this simulation the starting number of “Regulatory Cells” was increased (A; Initial Regulatory Cells = 2000) or decreased (B; Initial Regulatory Cells = 4). Efficient tumor control occurs either due to suppression of “Helper Cells” (A) or increased activity of “Killer Cells” (B).

The activity of “Helper Cells” and “Killer Cells” in the model is regulated by “Regulatory Cells.” With default values in the model, this regulation suppresses “Helper Cells” but at the same time suppresses “Killer Cells” which finally results in a tumor growth supporting micro-environment. Changing the number of “Regulatory Cells” can shift the environmental conditions in favor of the anti-tumor immune response. This can be achieved two ways (Fig 6). Increasing the number of “Regulatory Cells” (Initial Regulatory Cells = 2,000) suppresses the “Helper Cell” compartment and the subsequent lack of anti-apoptotic signals lead to inhibition of tumor growth (Fig 6A). On the other hand, decreasing the number of “Regulatory Cells” (Initial Regulatory Cells = 4) relieves the “Killer Cell” compartment that subsequently can eradicate the tumor cells (Fig 6B).

### Tumor growth with cytotoxic therapy

**Fig 7:**
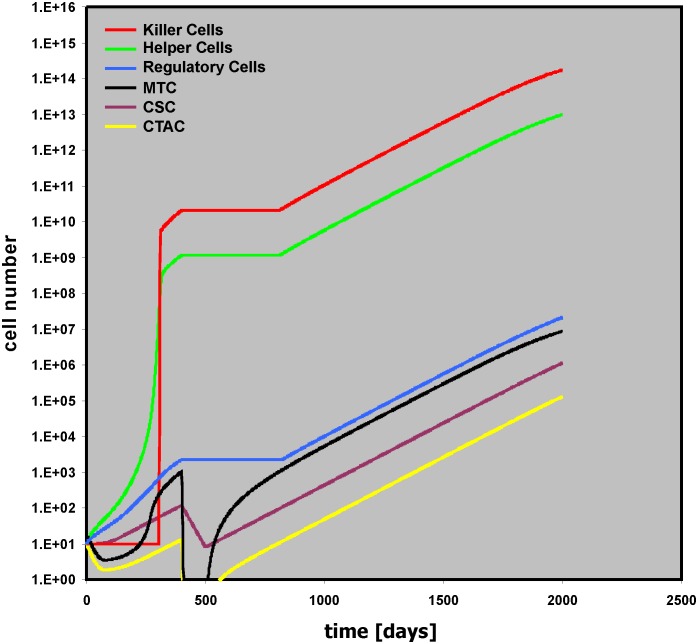
Relapse after insufficient therapeutic elimination of cancer stem cells. In this simulation, intensity of cytotoxic therapy against mature tumor cells and transit amplifying cells was increased (MTC THx intens = CTAC THx intens = 0.9), but intensity of cytotoxic therapy against cancer stem cells was low (CSC THx intens = 0.035). After the end of the therapy, tumor growth restarts.

Without therapeutic intervention, tumor growth is regulated by the activity of the micro-environment. We analyzed the effect of cytotoxic therapy (THx) in our model. Therapy with specificity for the mature tumor cells only (MTC THx intens = 0.9) leads only to a short slowdown of the tumor growth (S6 Fig). Tumor growth is stopped only after treatment that also affects the stem cell compartment of the tumor. Without toxicity for the bystander cells, large numbers of “Killer Cells,” “Helper Cells,” and “Regulatory Cells” remain present after therapy (S7 Fig). If toxicity for MTC and CTAC is high (MTC THx intens = CTAC THx intens = 0.9) but toxicity for the stem cell compartment is low (MTC THx intens = 0.035), tumor growth restarts after end of the Thx (Fig 7). Therapy usually has some toxicity for non-tumor cells.

**Fig 8:**
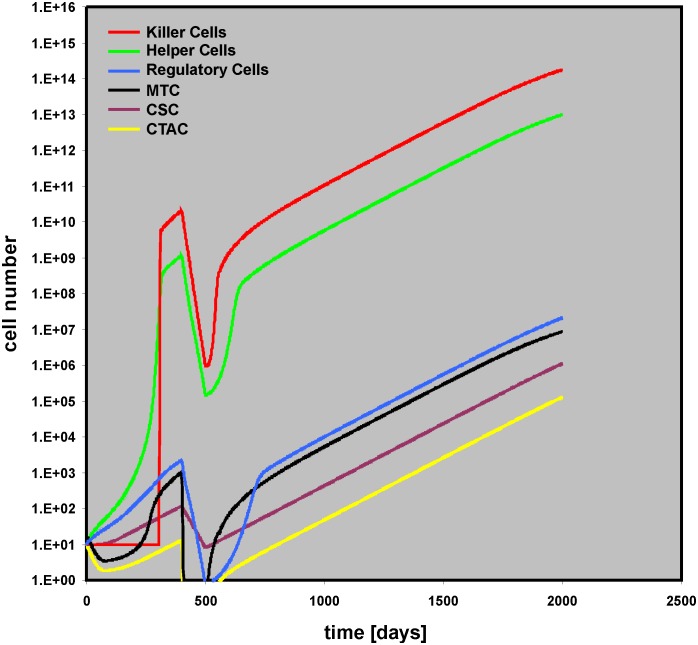
Relapse as a consequence of therapy related toxicity for “Killer Cells.” In this simulation, intensity of cytotoxic therapy against mature tumor cells and transit amplifying cells was high (MTC THx intens = CTAC THx intens = 0.9), but cytotoxic therapy against cancer stem cells was low (CSC THx intens = 0.035). Therapy related toxicity for bystander cells (Helper THx intens = Killer THx intens = Regulator THx intens = 0.1) did not allow tumor control by “Killer Cells” despite decreased activity of “Helper Cells.”

The model allows the simulation of these side-effects by using the variables “Helper THx intens,” “Killer THx intens,” and “Regulator THx intens.” Increased toxicity for “Helper Cells” (Helper THx intens = 0.1) suppresses anti-apoptotic signals from these cells and results in inhibition of tumor growth (S8 Fig). If the toxicity is the same for “Helper Cells,” Regulatory Cells,” and “Killer Cells,” the latter will no longer be able to kill tumor cells that have survived therapy and early relapse will occur (Fig 8).

**Fig 9:**
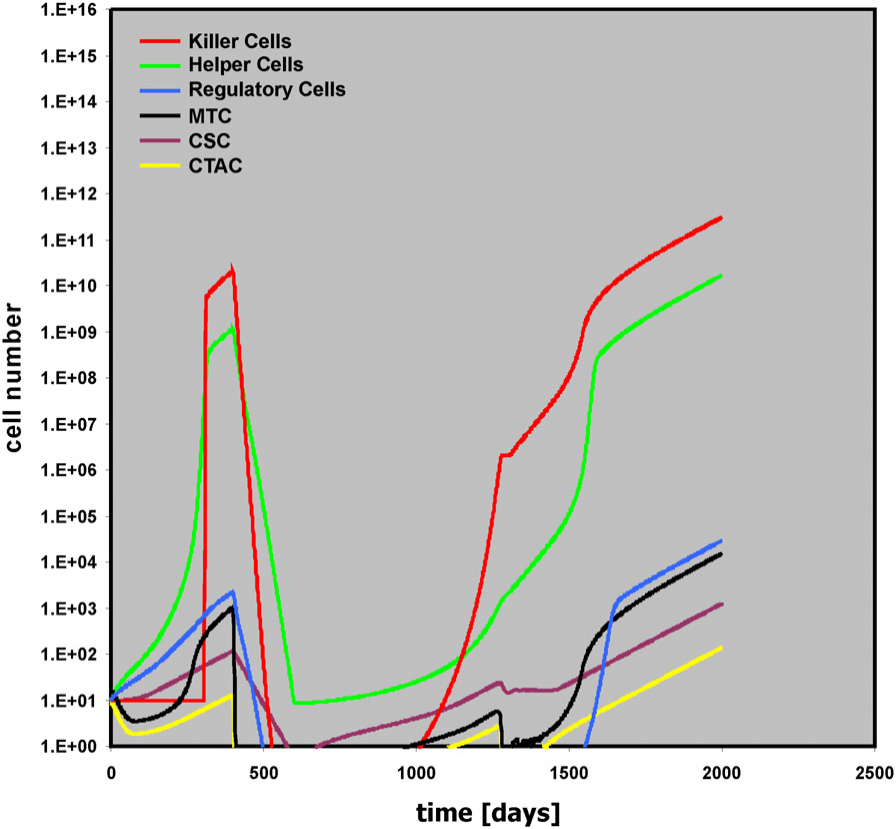
Late relapse due to increased therapy related toxicity for “Killer Cells.” In this simulation, the duration of cytotoxic therapy was increased from 100 to 200 days (for all cell types). Intensity of cytotoxic therapy against mature tumor cells and transit amplifying cells was high (MTC THx intens = CTAC THx intens = 0.9), but cytotoxic therapy against cancer stem cells was low (CSC THx intens = 0.035). Therapy related toxicity for “Killer Cells” was increased (Killer THx intensity = 0.2) compared with toxicity for other bystander cells (Helper THx intens = Regulator THx intens = 0.1).

Presence or absence of toxicity for “Regulatory Cells” has no impact on this behavior (S9 Fig). However, this toxicity for “Regulatory Cells” has a tremendous impact on outcome if the duration of the Thx is prolonged (THx dur (for all cell compartments) = 200): If the toxicity for all bystander cell compartments is the same (Helper THx intens = Killer THx intens = Regulator THx intens = 0.1), tumor growth stops (S10 Fig). Increasing the toxicity for “Killer Cells” only (Helper THx intens = Regulator THx intens = 0.1; Killer THx intens = 0.2), leads to relapse after remission (Fig 9).

**Fig 10:**
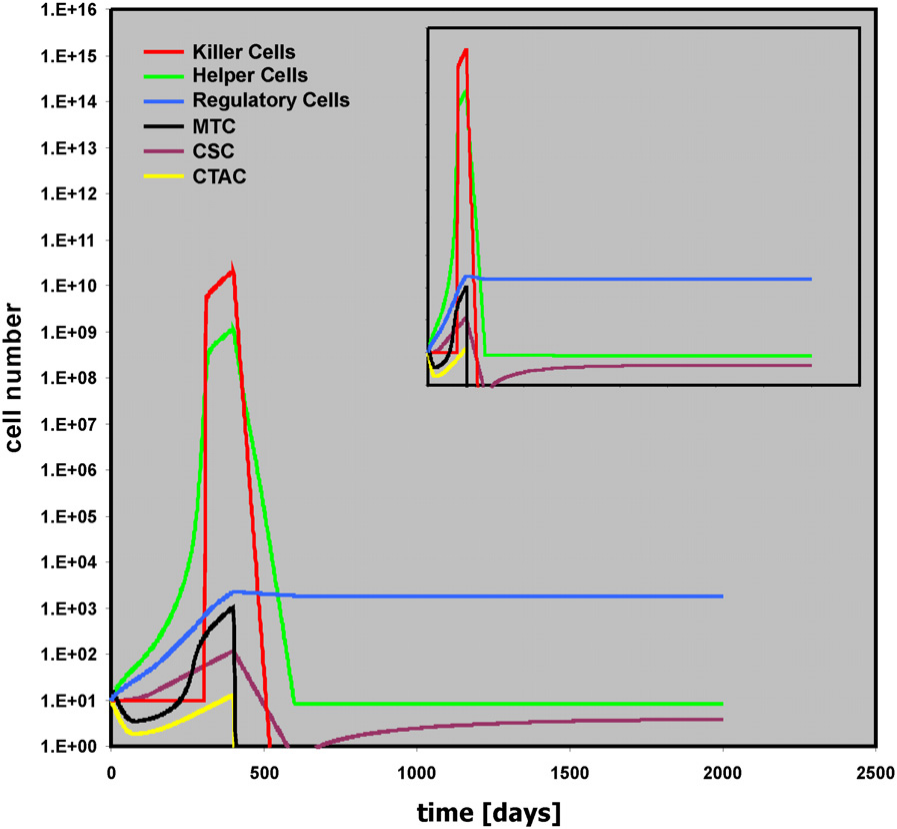
Stable remission due to decreased toxicity for “Regulatory Cells.” This simulation uses the same parameter values as described in the legend for Fig 9. However, therapy related toxicity for “Regulatory Cells” was lowered (Regulator THx intens = 0.001). The insert shows the simulation with a simulation time of 4,000 days.

On the other hand, if the toxicity for “Regulatory Cells” is reduced (Helper THx intens = 0.1; Killer THx intens = 0.2; Regulator THx intens = 0.001), long lasting remission is achieved (Fig 10). An interesting feature of this situation is that with slightly higher toxicities for “Regulatory Cells” (Helper THx intens = 0.1; Killer THx intens = 0.2; Regulator THx intens = 0.01) very late relapses can be observed (S11 Fig).

### Immunotherapy (ImmunoThx)-extensions of the basic model

**Fig 11:**
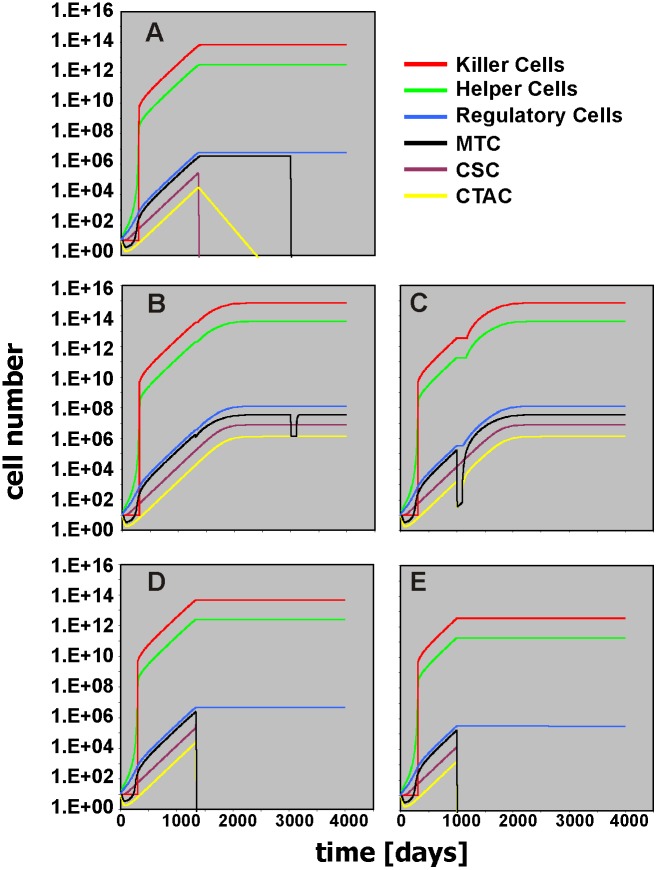
Paradox tumor enhancement after reduction of mature tumor cells. In these simulations, killing activity against cancer stem cells was increased (ImmunoTHx2CSC intens = 53). This increase leads to stable tumor control with elimination of cancer stem cells. Remaining tumor cells can successfully be eliminated by subsequent cytotoxic therapy (MTC THx intens = 0.9, MTC THx time = 3000) (A). Additional increase in the activity of “Killer Cells” against mature tumor cells and transit amplifying cells (ImmunoTHx2MTC intens = ImmunoTHx2CTAC intens = 53) lead to paradox increase in tumor cell number (B). Under these conditions, cytotoxic therapy (MTC THx intens = 0.9, MTC THx time = 3000) cannot eradicate tumor cells. Similar effects were observed after ill-timed cytotoxic therapy (MTC THx intens = CTAC THx intens = 0.9; MTC THx time = CTAC THx time = 1000) against MTC and CTAC (C). In both cases, increasing the number of “Killer Cells” (ImmunoTHx1 time = 1000; ImmunoTHX1 intens = 3.5e+010) restored tumor control (D: ImmunoTHx2MTC intens = ImmunoTHx2CTAC intens = 53; E: CTAC THx intens = MTC THx intens = 0.9; CTAC THx time = MTC THx time = 1000). The time scale for all simulations in this figure is 4000 days.

The model can be used for the simulation of immunotherapeutic interventions. The first type of immunotherapy that can be simulated (ImmunoThx1) is adoptive cell transfer (S12 Fig). With this immunotherapy the number of “Killer Cells” can be increased (simulating adoptive transfer of “Killer Cells”) starting at time point ImmunoTHx1 time (default value = 300), lasting for ImmunoTHx1 dur (default value = 1), and with intensity ImmunoTHx1 intens (default value = 0). At early stages of tumor development, the outcome of slightly increasing the number of “Killer Cells” (ImmunoTHx intens = 1e+006) depends on the precise time point of ImmunoTHx1 (S13 Fig). Whereas ImmunoThx1 before day 202 and between day 244 and 291 suppresses tumor growth, tumor growth is not suppressed between day 202 and day 243. This immune-escape window is a consequence of the different growth kinetics of CSC, MTC, and CTAC that allows complete elimination of tumor cells or not. Due to the lack of high “Helper Cell” activity at early stages of tumor growth, the numbers of MTC and CTAC decrease initially (see also Fig 4). Consequently, if the number of initial MTC and CTAC is set to zero, the immune-escape window disappears. At day 300, ImmunoTHx intens of 1.5e+010 eliminates the tumor. At day 302 this is no longer possible. In this case, increasing the duration of the therapy (ImmunoTHx1 dur = 2) leads to tumor elimination (S14 Fig). At later time points, increased numbers of “Killer Cells” have to be added in order to achieve therapeutic effects: at day 500 more than 10^12^ “Killer Cells” are required, at day 1,000 more than 10^14^, and on day 1,500 more than 10^15^ (S15 Fig). Obviously, the low cytotoxic activity of these cells hinders efficient tumor control. Immunotherapeutic interventions aim not only at increasing the number of effector cells (*e*.*g*. by adoptive transfer) but also at increasing the activity of these cells. Therefore, we included a second ImmunoTHx modifier in the model (S16 Fig). This part of the model allows to increase the killing activity of the “Killer Cells.” If the increment is high, tumor growth is suppressed (S17 Fig). Increasing the killing activity against CSC is sufficient for this suppression (S17 Fig). In contrast, the killing of tumor cells without killing CSC did not suppress tumor growth (S17 Fig). An interesting situation occurs if killing of CSC is sufficient but no more than sufficient (ImmunoTHx2CSC intens = 53). In this situation, increased killing of CTAC and MTC can paradoxically favor tumor growth (Fig 11). Cytotoxic therapy has the same effect in the model (Fig 11). The reason for this behavior is the lack of stimulation of additional “Killer Cells” due to the loss of stimulatory tumor cells. Consequently, increasing the number of “Killer Cells” by ImmunoTHx1 can re-adjust the balance in favor of the immune system (Fig 11). Depletion of helper cells has a similar effect (S18 Fig). Interestingly, a short-term increase of the number of regulatory cells at the time point of ImmunoTHx2 (simulated by Regulator THx intens = -0.9) can induce tumor suppression (S19 Fig). A short-term inhibition of killer cells has the same effect (S19 Fig). The reason for this behavior is the fact that with increased killing capacity the number of tumor cells decreases below a threshold for sufficient stimulation of “Killer Cells.” Decreasing the number of “Killer Cells” in this sensitive phase can increase the number of tumor cells above this threshold, followed by efficient stimulation of “Killer Cells” and elimination of tumor cells.

The aim of this study was the generation of a model that simulates essential features of Hodgkin’s lymphoma which is characterized of a usually small number of tumor cells that stimulate a high number of bystander cells which in turn generate a micro-environment that allows tumor growth. The micro-environment of HL is highly complex and composed of many more cell types than have been included in our model. However, even after reduction of this complexity to three principle components (“Helper Cells,” “Killer Cells,” “Regulatory Cells”), the model allows the simulation of different outcomes (*e*.*g*., Fig 4: progressive disease; Fig 7: early relapse; Fig 9: late relapse; Fig 10: continuous remission). Based on the results from this model, one can expect that (immunological) treatment can have surprising and paradox results if the treatment changes the ratio between tumor promoting and tumor rejecting activities or changes the immune-stimulatory activity of the tumor. Under certain conditions, therapy can enhance tumor growth. The model is not restricted to Hodgkin’s lymphoma but might be able to predict the behavior of other tumors with strong interaction between tumor cells and stromal cells. Decreased activity of tumor-specific cytotoxic T can be associated with improved tumor control *in vivo* [15]. Enhancement of tumor growth has been observed in experimental model for cancer immunotherapy and faster tumor growth has been anecdotally observed after immunotherapy in humans [16, 17]. In addition to classical antibody-based immunological enhancement [18], regulatory interactions between different cell types can explain such effects. Twenty years ago, R. T. Prehn suggested that under certain conditions immunosuppression can lead to tumor remission [19]. Indeed, our simple model demonstrates that the paradoxical outcome of immunotherapy can be enhanced tumor growth and that immunosuppression can lead to elimination of tumors.

If treatment intensity is high enough, tumor growth can be suppressed completely but treatment-related toxicity hinders the unlimited intensification of treatment. This toxicity can lead to very late side effects. For example, development of secondary malignancies can occur in patients treated for HL [20,21]. Therefore, the reduction of treatment-related toxicity is a major objective of ongoing clinical trials. One of the predictions from the proposed model is that under conditions of lower treatment intensity, treatment-related toxicity can be involved in regulation of anti-tumor effects. In the future, optimal targeting of the micro-environment including cells with regulatory function finally leading either to inhibition of cells with helper activity or to stimulation of cells with killer activity can allow further reduction of cytotoxic therapy. The proposed model might be useful for *in-silico* analysis of different scenarios that have to be further investigated in experimental models.

In the present implementation of the model, the tumor micro-environment includes only three different cell populations. However, the HL micro-environment contains far more cell types which might influence the behavior of the system. This limitation in mind, the present model can be useful for basic analysis, and additional components might be added for optimized simulation of the behavior of the highly multi-component HL micro-environment.

## Supporting Information

S1 Model This file contains the model (see Fig 3) in Vensim (vpm) format. This file can be used in conjunction with Vensim Model Reader (http://vensim.com/vensim-model-reader/) for running the model with altered starting conditions.
(VPM)

S1 Fig Absence of tumor growth due to absence of initial CSC. In this simulation, the number of initial cancer stem cells was set to zero. For all other parameters default values were used.
(TIF)

S2 Fig Reduced tumor growth due to absence of proliferation of CSC. In this simulation, the growth rate of cancer stem cells was set to zero. For all other parameters default values were used.
(TIF)

S3 Fig Complete absence of tumor growth due to absence of proliferation of CSC in combination with absence of proliferation of CTAC or increased activity of “Killer Cells.” In this simulation, the growth rate of cancer stem cells was set as zero. In addition, the growth rate of transit amplifying cells was set as zero (A) or the killing activity of “Killer Cells” for mature tumor cells was increased to 5e-006 (B). For all other parameters default values were used.
(TIF)

S4 Fig Absence of tumor growth due to absence of initial “Helper Cells.” In this simulation, the number of initial “Helper Cells” was set to zero. For all other parameters default values were used.
(TIF)

S5 Fig Absence of tumor growth due to increased CSC-killing potential of “Killer Cells.” In this simulation, the killing activity of “Killer Cells” for cancer stem cells was increased (Kill of CSC = 0.1). For all other parameters default values were used.
(TIF)

S6 Fig Insufficient therapy due to lack of CSC targeting. In this simulation, therapy was simulated with MTC THx intens = 0.9. For all other parameters default values were used.
(TIF)

S7 Fig Successful therapy due to CSC targeting. In this simulation, therapy was simulated with MTC THx intens = CSC THx intens = CTAC THx intens = 0.9. For all other parameters default values were used.
(TIF)

S8 Fig Successful therapy due to targeting of “Helper Cells.” In this simulation, therapy was simulated with MTC THx intens = CTAC THx intens = 0.9, CSC THx intens = 0.035, and Helper THx intens = 0.1. For all other parameters default values were used.
(TIF)

S9 Fig Relapse due to therapy-related toxicity for “Killer Cells.” In this simulation, therapy-related toxicity was simulated with Killer THx intens = 0.1. For all other parameters the same values were used as in S8 Fig.
(TIF)

S10 Fig Tumor eradication after increased treatment time. In this simulation, therapy duration was set to 200 for all cell types. In addition, therapy-related toxicity was simulated with Regulator THx intens = 0.1. For all over parameters the same values were used as in S9 Fig. After end of therapy, spontaneous regression of a small relapse can be seen.
(TIF)

S11 Fig Late relapse due to therapy-related toxicity. In this simulation, therapy-related toxicity was simulated with Regulator THx intens = 0.01 and Killer THx intens = 0.2. For all other parameters the same values were used as in S10 Fig. The insert shows the simulation with a simulation time of 4000 days.
(TIF)

S12 Fig First immunotherapy extension of the model. With this extension (adoptive transfer of “Killer Cells”), adoptive immunotherapy (ImmunoTHx1) can be simulated. ImmunoTHx1 increases the number of “Killer Cells” by the factor ImmunoTHx1 intens at time point ImmunoTHx1 time. The parameter ImmunoTHx1 dur can be used for simulation of multiple doses of “Killer Cells.”
(TIF)

S13 Fig The success of adoptive immunotherapy depends on the time point of treatment. In this simulation, adoptive immunotherapy was simulated by ImmunoTHx intens = 1e+006 and ImmunoTHx dur = 1. Therapy was started at three different time points.
(TIF)

S14 Fig The success of adoptive immunotherapy depends on the time point of treatment and the intensity of treatment. In this simulation, adoptive immunotherapy was simulated by ImmunoTHx1 intens = 1.5e+010. Therapy was started at two different time points. The duration of the therapy was set as 1 or 2 (simulating lower or higher number of single doses of adoptively transferred “Killer Cells”).
(TIF)

S15 Fig The success of adoptive immunotherapy depends on the time point of treatment and the intensity of treatment. In this simulation, adoptive immunotherapy was simulated by different combinations of ImmunoTHx1 intens (1e+012–1e+016) and ImmunoTHx1 time (500–1,500). The duration of the therapy was set as 1 for all combinations. The red line indicates conditions with tumor growth.
(TIF)

S16 Fig Second immunotherapy extension of the model. With this extension (increasing the killing activity of “Killer Cells”), the effect of increasing the killing activity of “Killer Cells” (*e*.*g*. by vaccination) can be simulated (ImmunoTHx2). ImmunoTHx2 increases the killing capacity of “Killer Cells” for all the types of tumor cells independently. In addition, the time point for ImmunoThx2 as well as the duration of the effect can be set individually for all tumor cell types. With the default values for the duration, killing activity remains stable for 3000 days of the simulation.
(TIF)

S17 Fig Success of immunotherapy depends on targeting of CSC. In these simulations, different combinations of ImmunoTHx2CSC intens, ImmunoTHx2CTAC intens and ImmunoTHx2MTC intens were used. Tumor growth stops only after sufficient increase of the killing activity against CSC. The time scale for all simulations in this figure is 4000 days.
(TIF)

S18 Fig Increased efficiency of immunotherapy in combination with elimination of “Helper Cells.” In these simulations, immunotherapy was simulated with ImmunoTHx2CSC intens = ImmunoTHx2CTAC = ImmunoTHx2MTC = 53. This sub-optimal therapy (see also S19 Fig) can be rendered successful by decreasing the number of “Helper Cells” by a “Helper Cell”-specific therapy (Helper THx time = 1000; Helper THX intens = 0.7). The time scale for this simulation is 4000 days.
(TIF)

S19 Fig Increased efficiency of immunotherapy in combination with elimination of “Killer Cells” or increase of “Regulatory Cells.” In these simulations, immunotherapy was simulated with ImmunoTHx2CSC intens = ImmunoTHx2CTAC intens = ImmunoTHx2MTC intens = 53. This sub-optimal therapy (upper panel) can be rendered successful by a short pulse of toxic therapy for “Killer Cells” (Killer THx time = 1000; lower panel) or an increase of the number of “Regulatory Cells” (Regulator THx time = 1000; middle panel). The time scale for all simulations in this figure is 4000 days.
(TIF)
