# Supplementary figures and images for: A Multi-Component Model of Hodgkin's Lymphoma
Source: PLoS One. 2015 Apr 27;10(4):e0124614. doi: 10.1371/journal.pone.0124614 (PMC4411114; doi:10.1371/journal.pone.0124614)

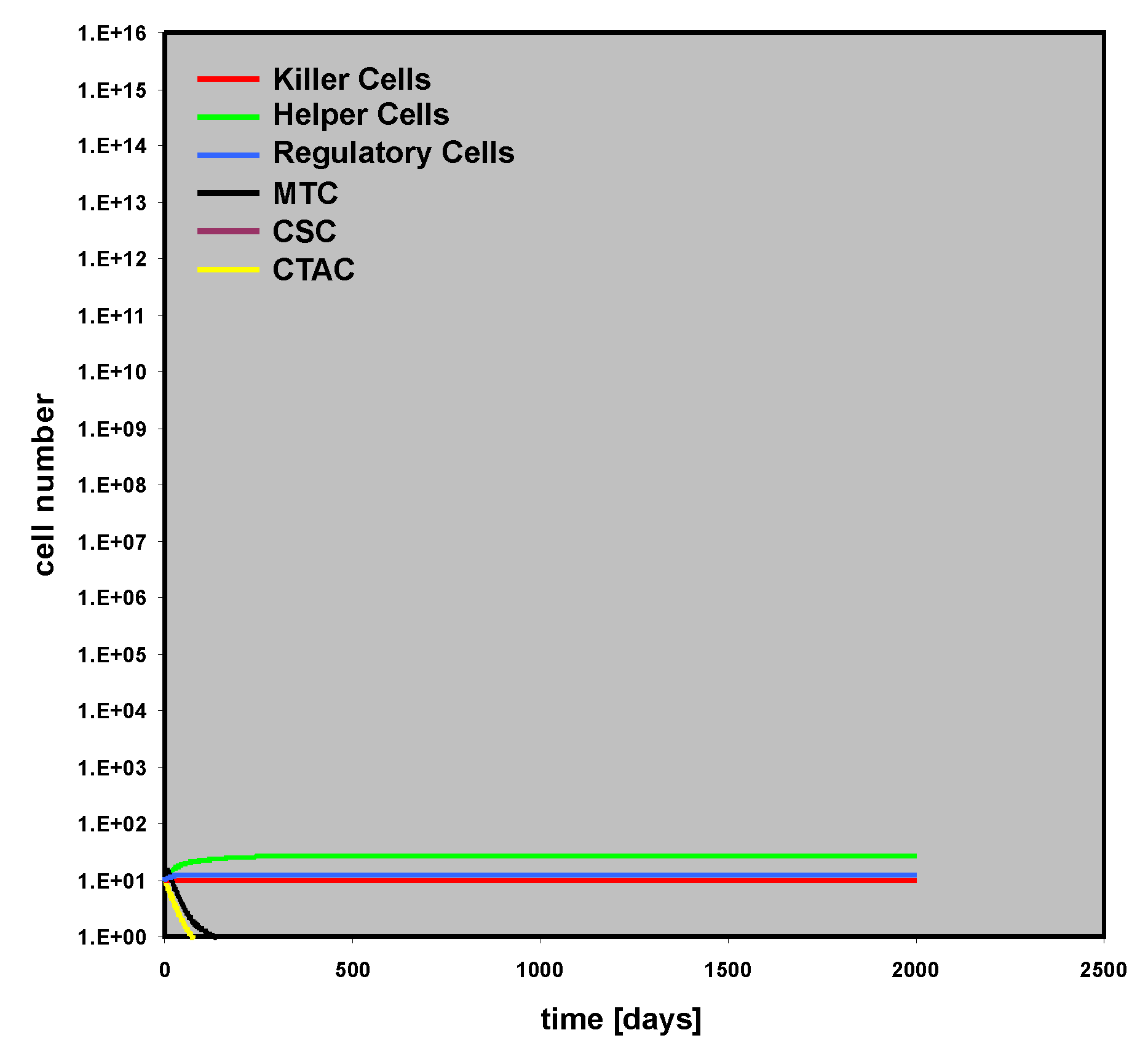

Supplement: S1 Fig — In this simulation, the number of initial cancer stem cells was set to zero. For all other parameters default values were used. (TIF) [file pone.0124614.s002.tif]

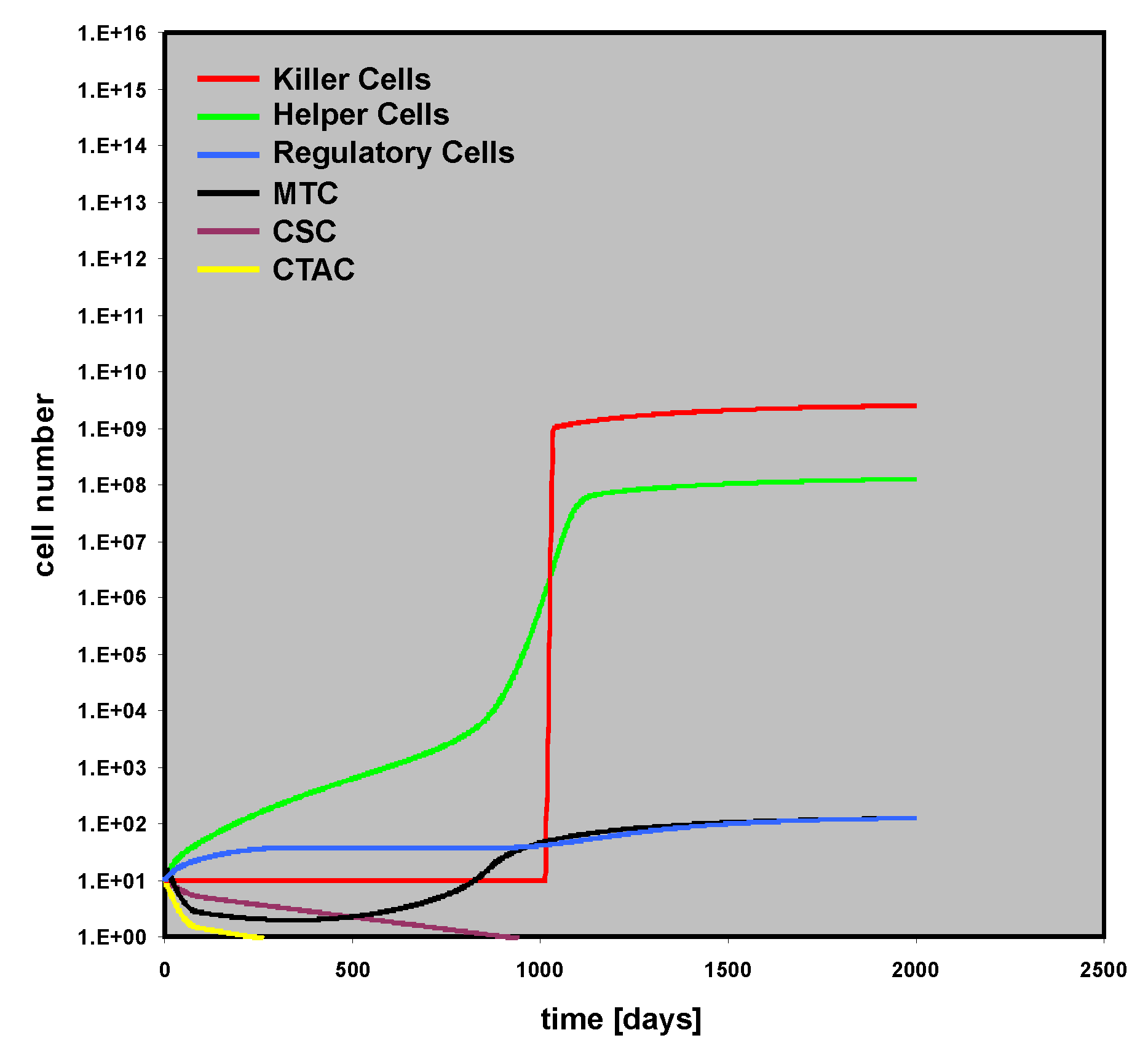

Supplement: S2 Fig — In this simulation, the growth rate of cancer stem cells was set to zero. For all other parameters default values were used. (TIF) [file pone.0124614.s003.tif]

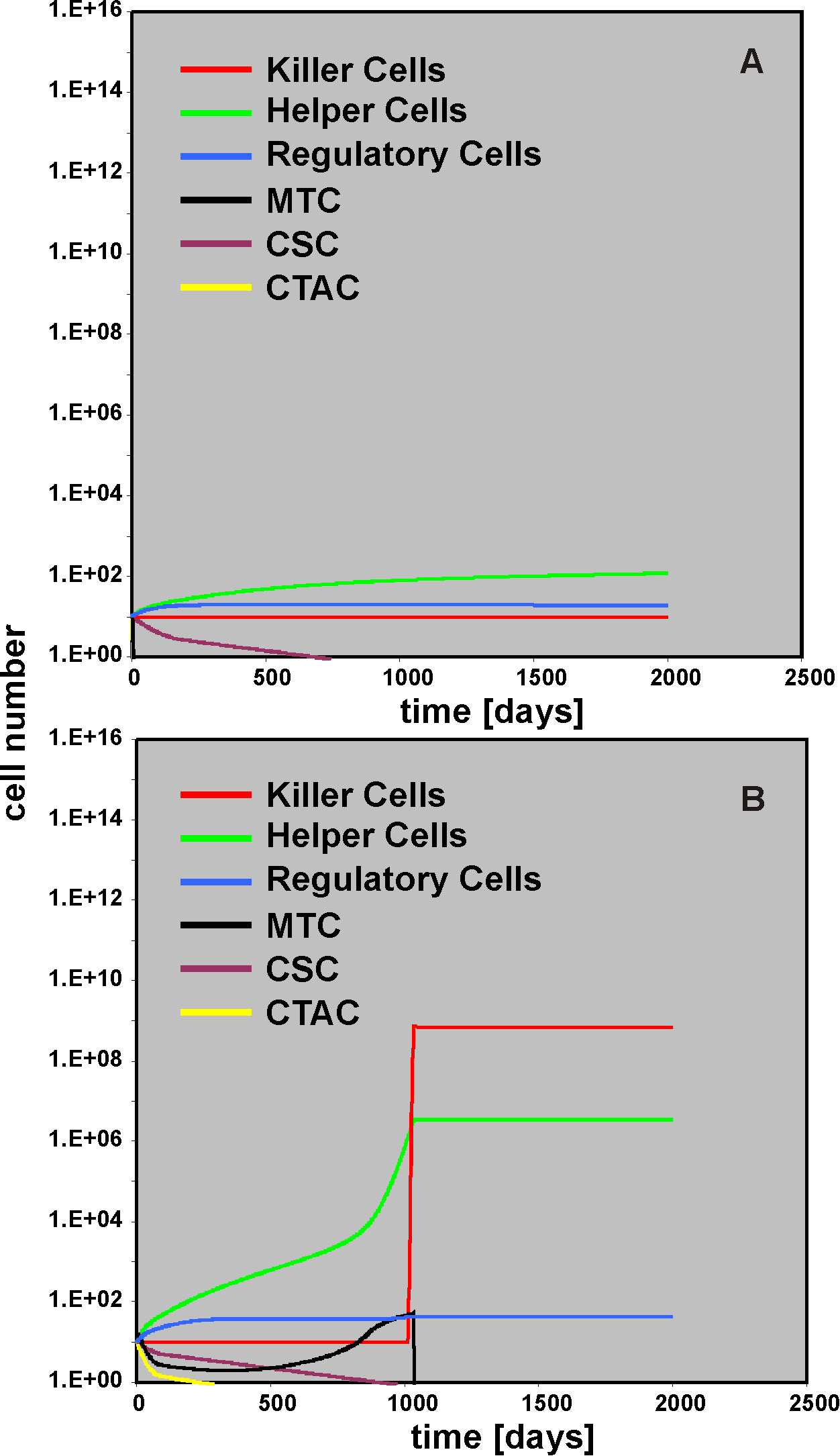

Supplement: S3 Fig — In this simulation, the growth rate of cancer stem cells was set as zero. In addition, the growth rate of transit amplifying cells was set as zero (A) or the killing activity of “Killer Cells” for mature tumor cells was increased to 5e-006 (B). For all other parameters default values were used. (TIF) [file pone.0124614.s004.tif]

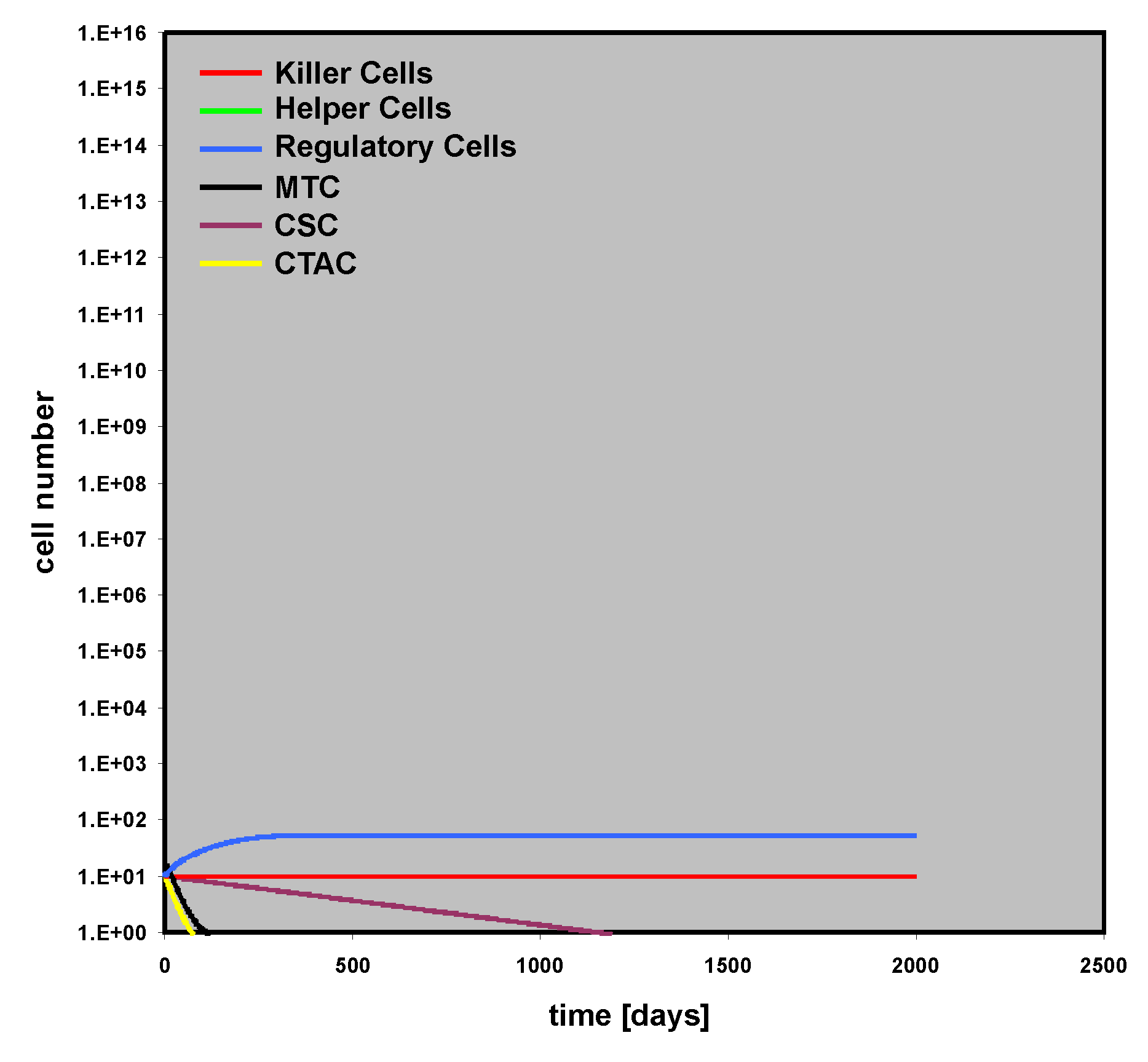

Supplement: S4 Fig — In this simulation, the number of initial “Helper Cells” was set to zero. For all other parameters default values were used. (TIF) [file pone.0124614.s005.tif]

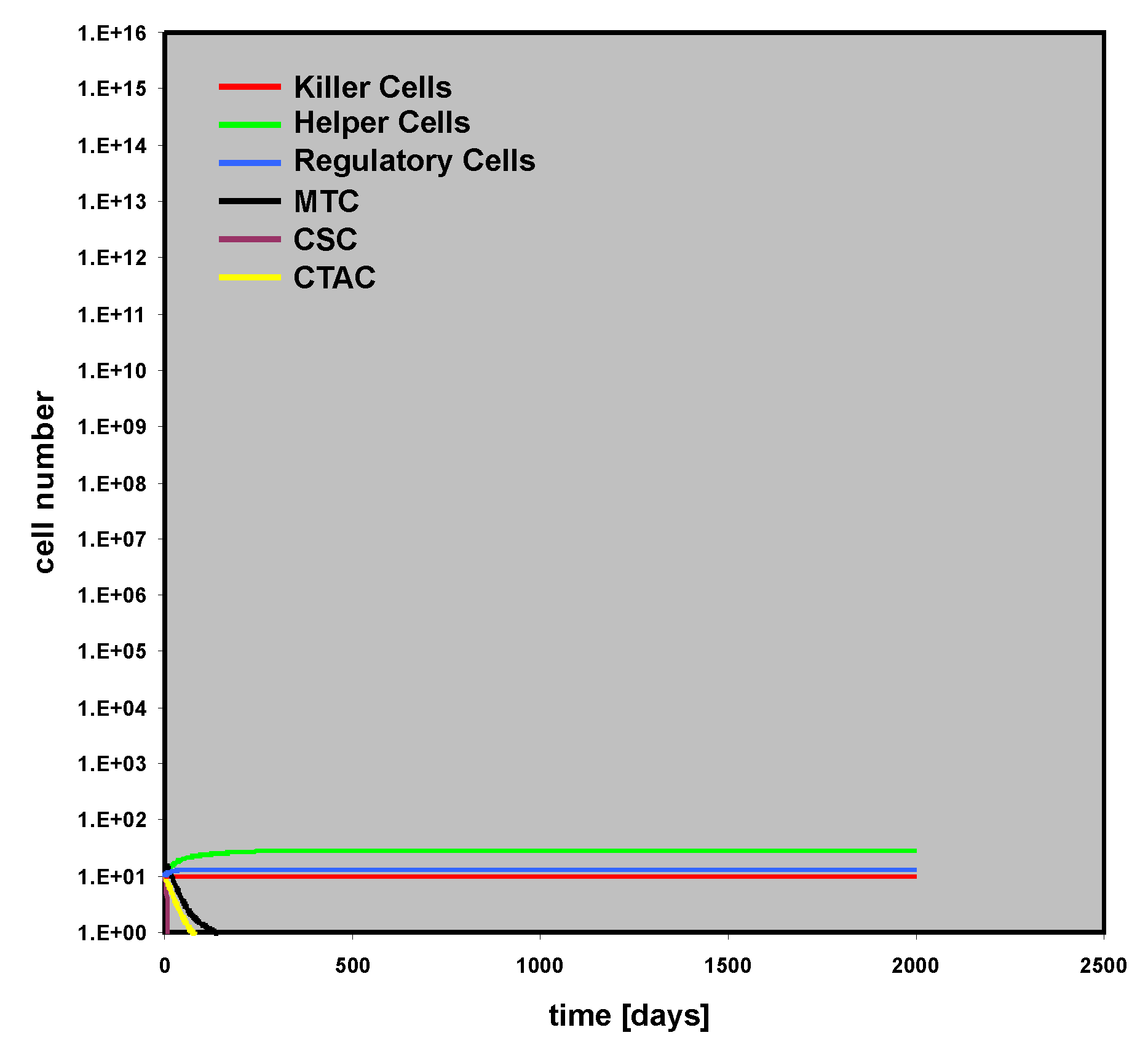

Supplement: S5 Fig — In this simulation, the killing activity of “Killer Cells” for cancer stem cells was increased (Kill of CSC = 0.1). For all other parameters default values were used. (TIF) [file pone.0124614.s006.tif]

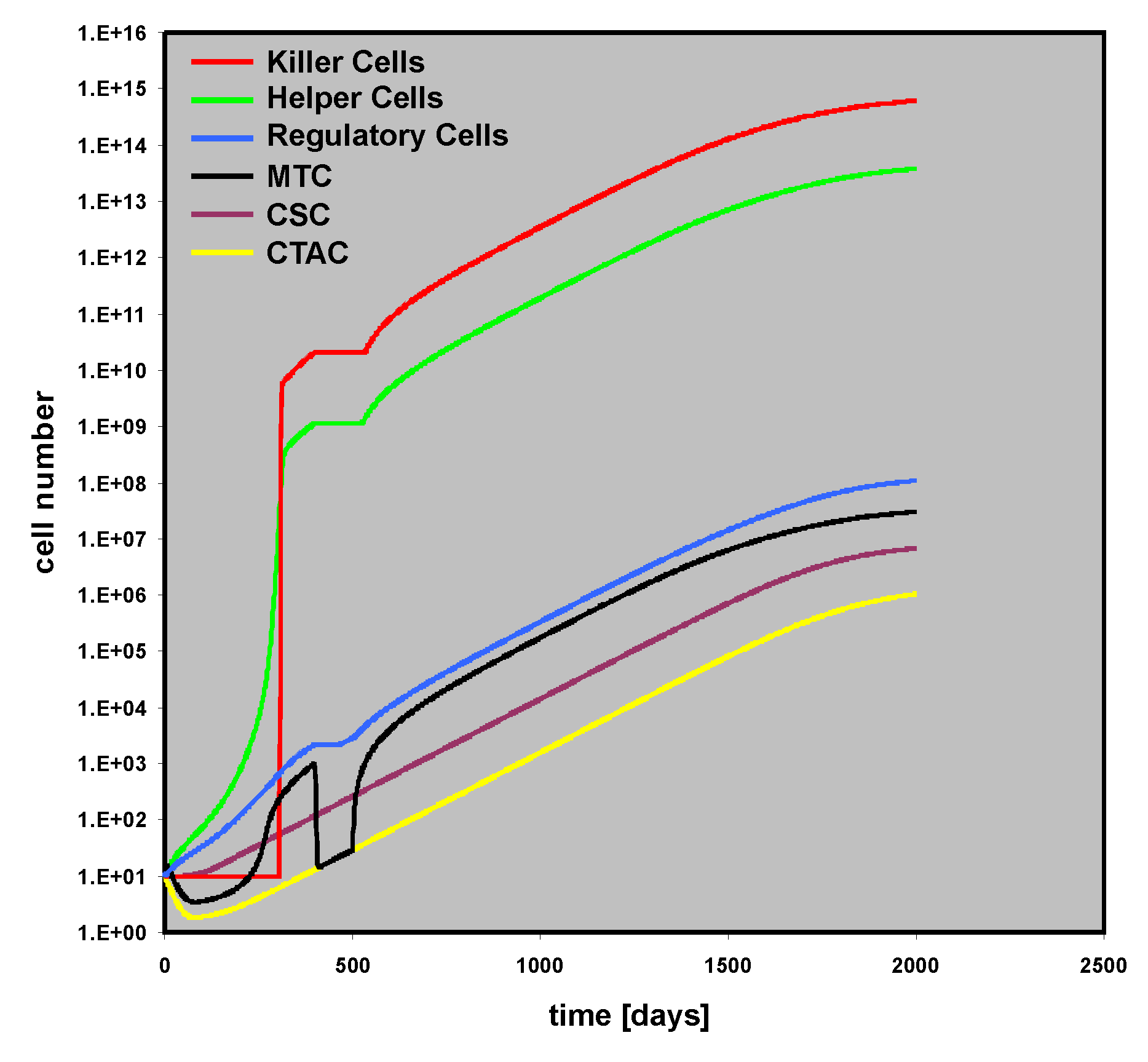

Supplement: S6 Fig — In this simulation, therapy was simulated with MTC THx intens = 0.9. For all other parameters default values were used. (TIF) [file pone.0124614.s007.tif]

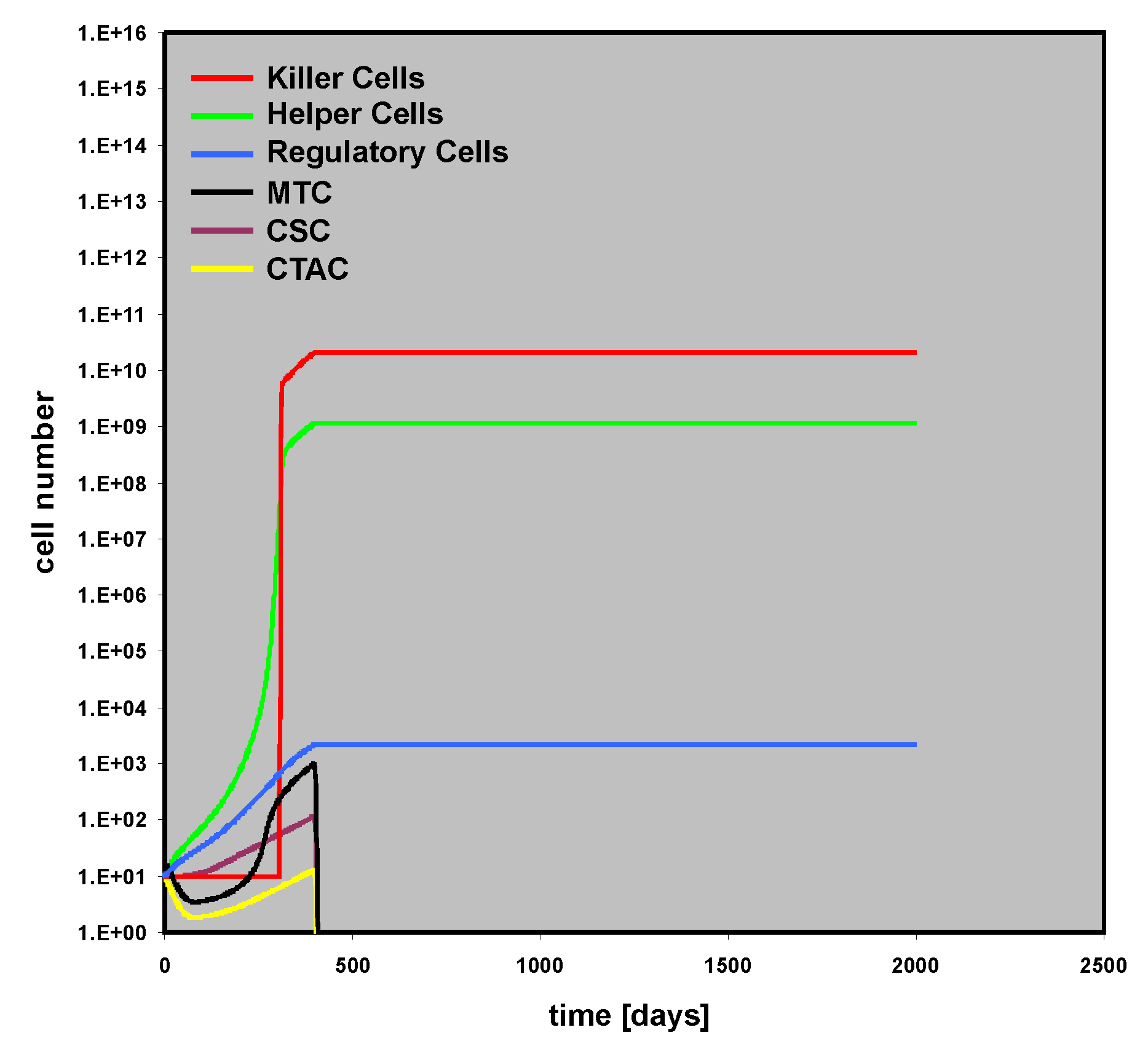

Supplement: S7 Fig — In this simulation, therapy was simulated with MTC THx intens = CSC THx intens = CTAC THx intens = 0.9. For all other parameters default values were used. (TIF) [file pone.0124614.s008.tif]

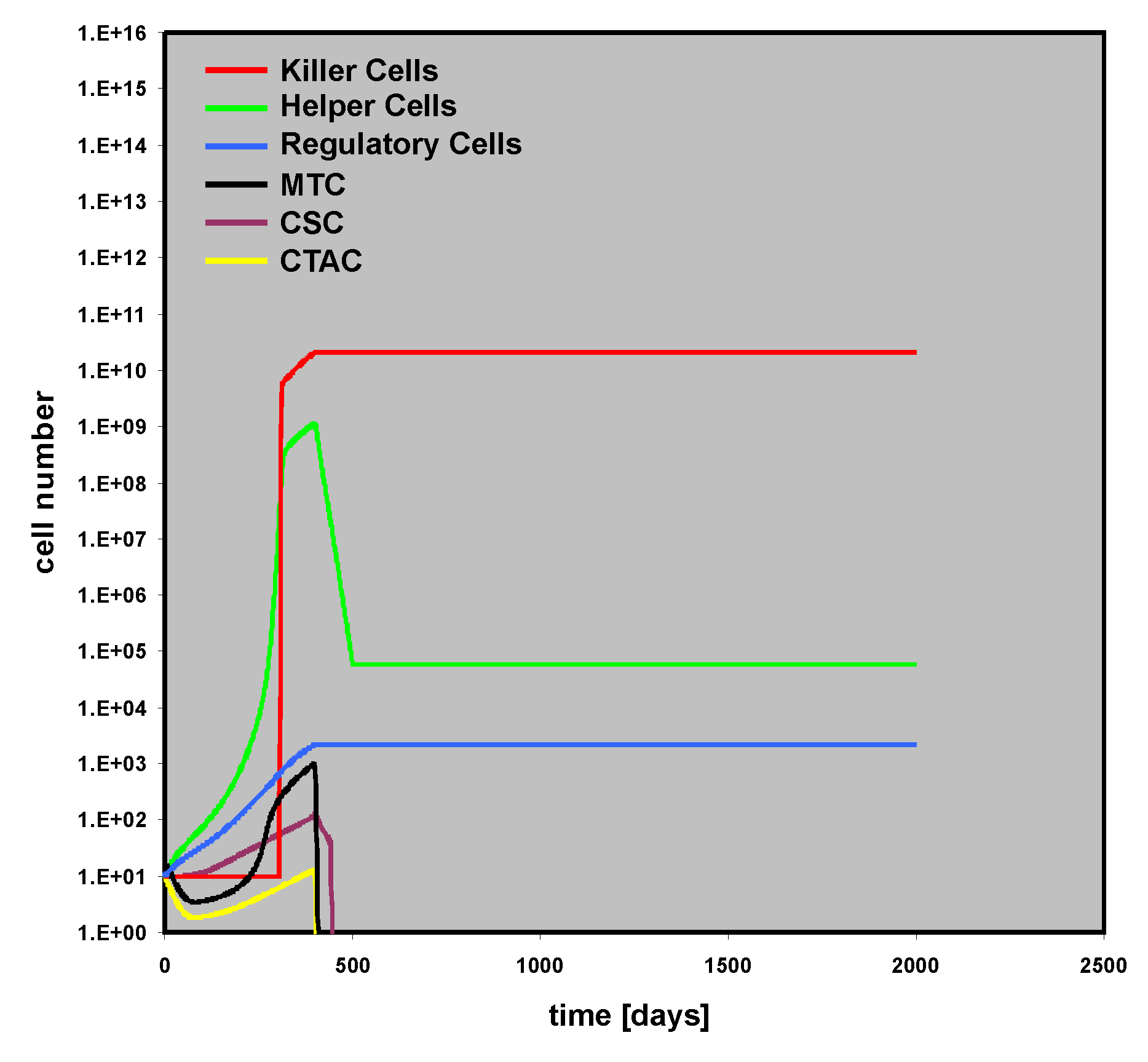

Supplement: S8 Fig — In this simulation, therapy was simulated with MTC THx intens = CTAC THx intens = 0.9, CSC THx intens = 0.035, and Helper THx intens = 0.1. For all other parameters default values were used. (TIF) [file pone.0124614.s009.tif]

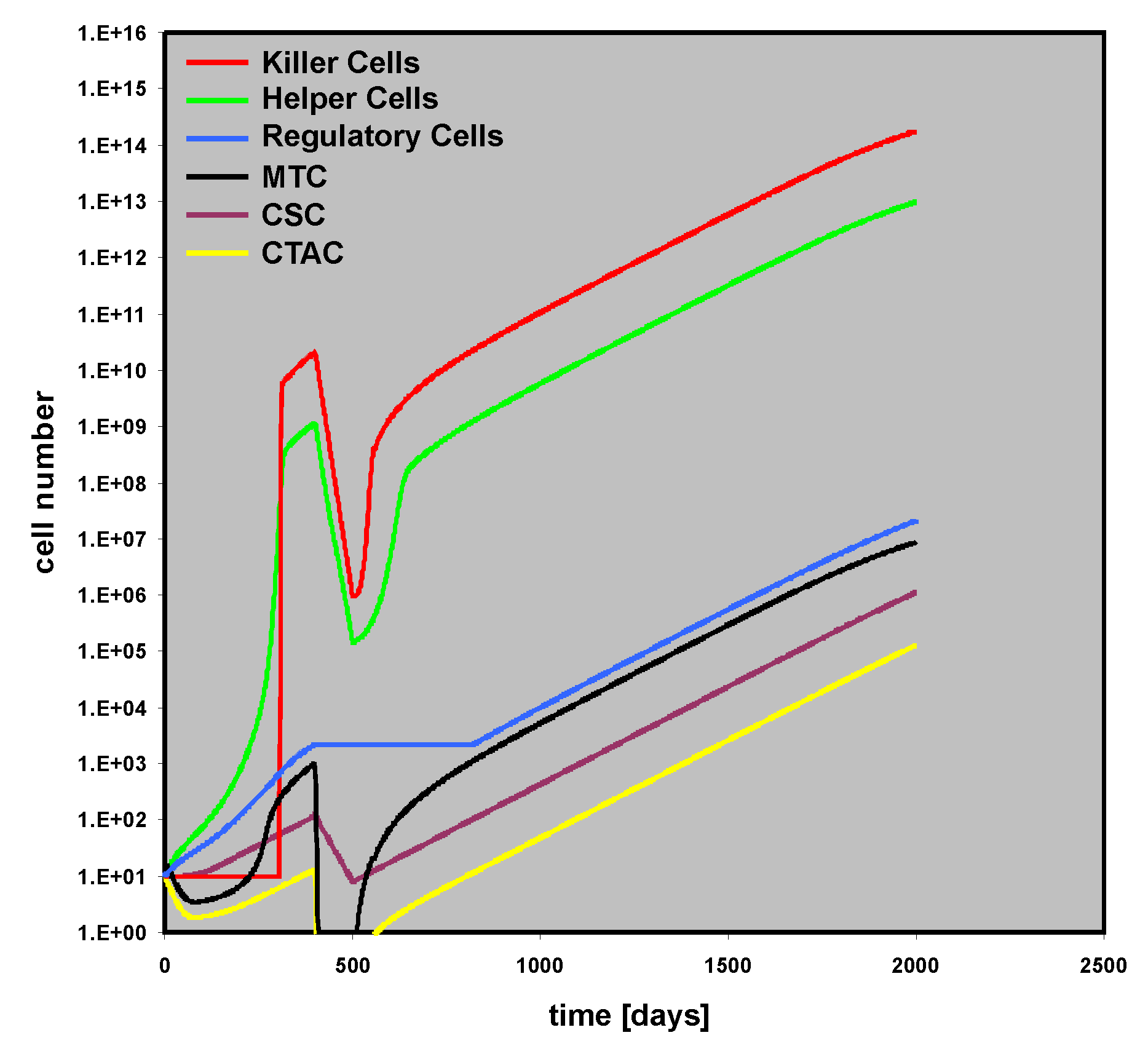

Supplement: S9 Fig — In this simulation, therapy-related toxicity was simulated with Killer THx intens = 0.1. For all other parameters the same values were used as in S8 Fig. (TIF) [file pone.0124614.s010.tif]

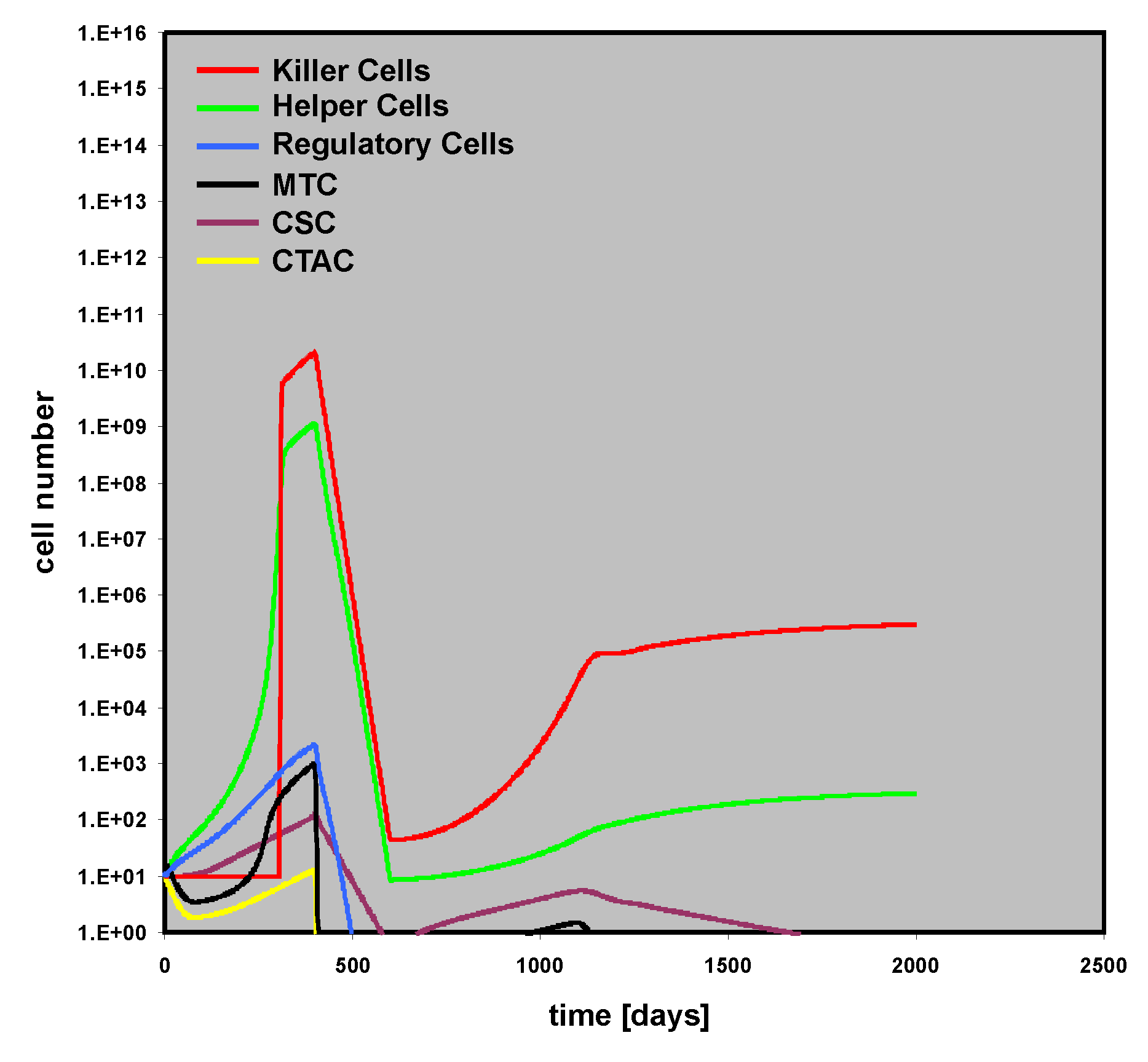

Supplement: S10 Fig — In this simulation, therapy duration was set to 200 for all cell types. In addition, therapy-related toxicity was simulated with Regulator THx intens = 0.1. For all over parameters the same values were used as in S9 Fig. After end of therapy, spontaneous regression of a small relapse can be seen. (TIF) [file pone.0124614.s011.tif]

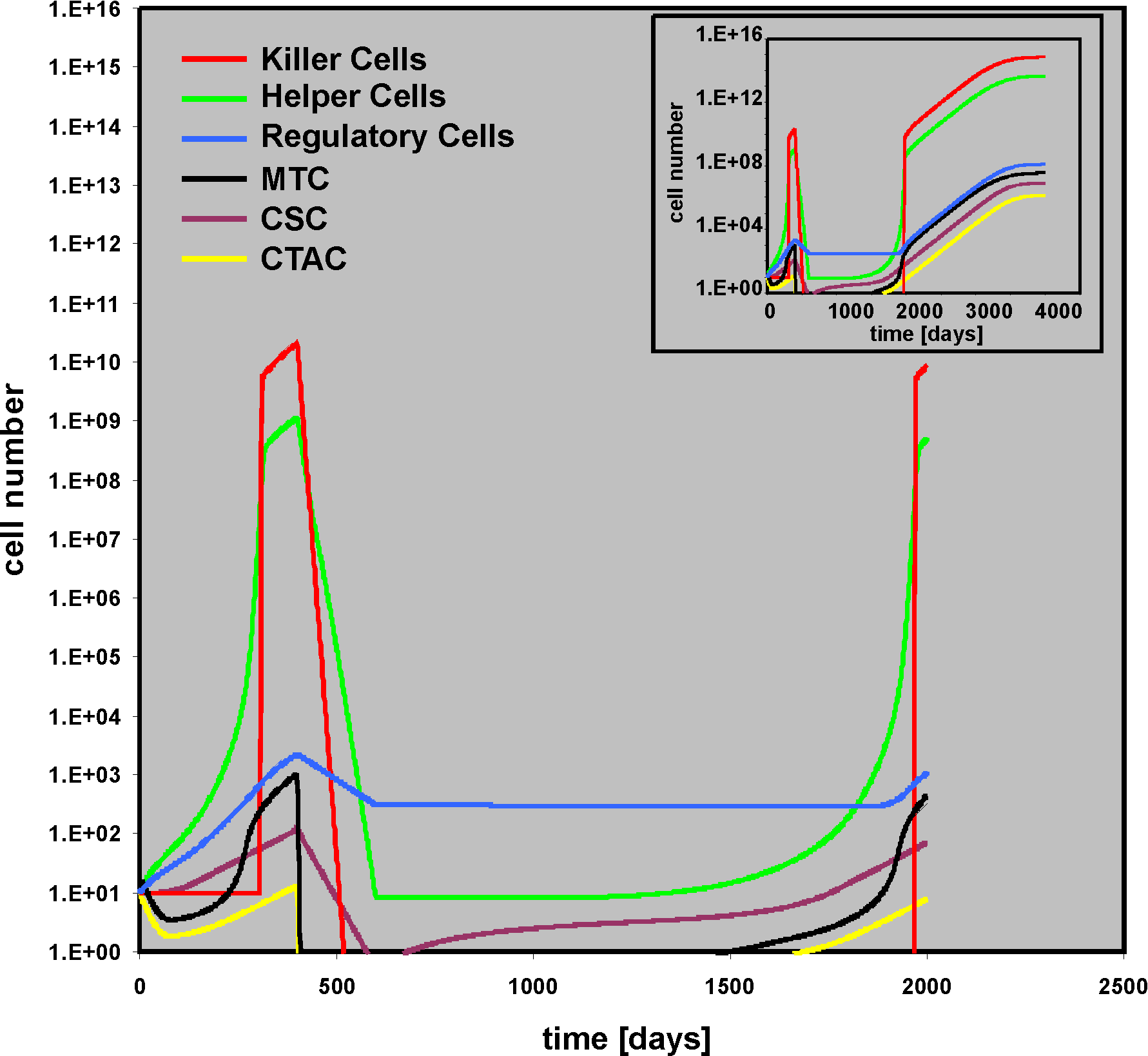

Supplement: S11 Fig — In this simulation, therapy-related toxicity was simulated with Regulator THx intens = 0.01 and Killer THx intens = 0.2. For all other parameters the same values were used as in S10 Fig. The insert shows the simulation with a simulation time of 4000 days. (TIF) [file pone.0124614.s012.tif]

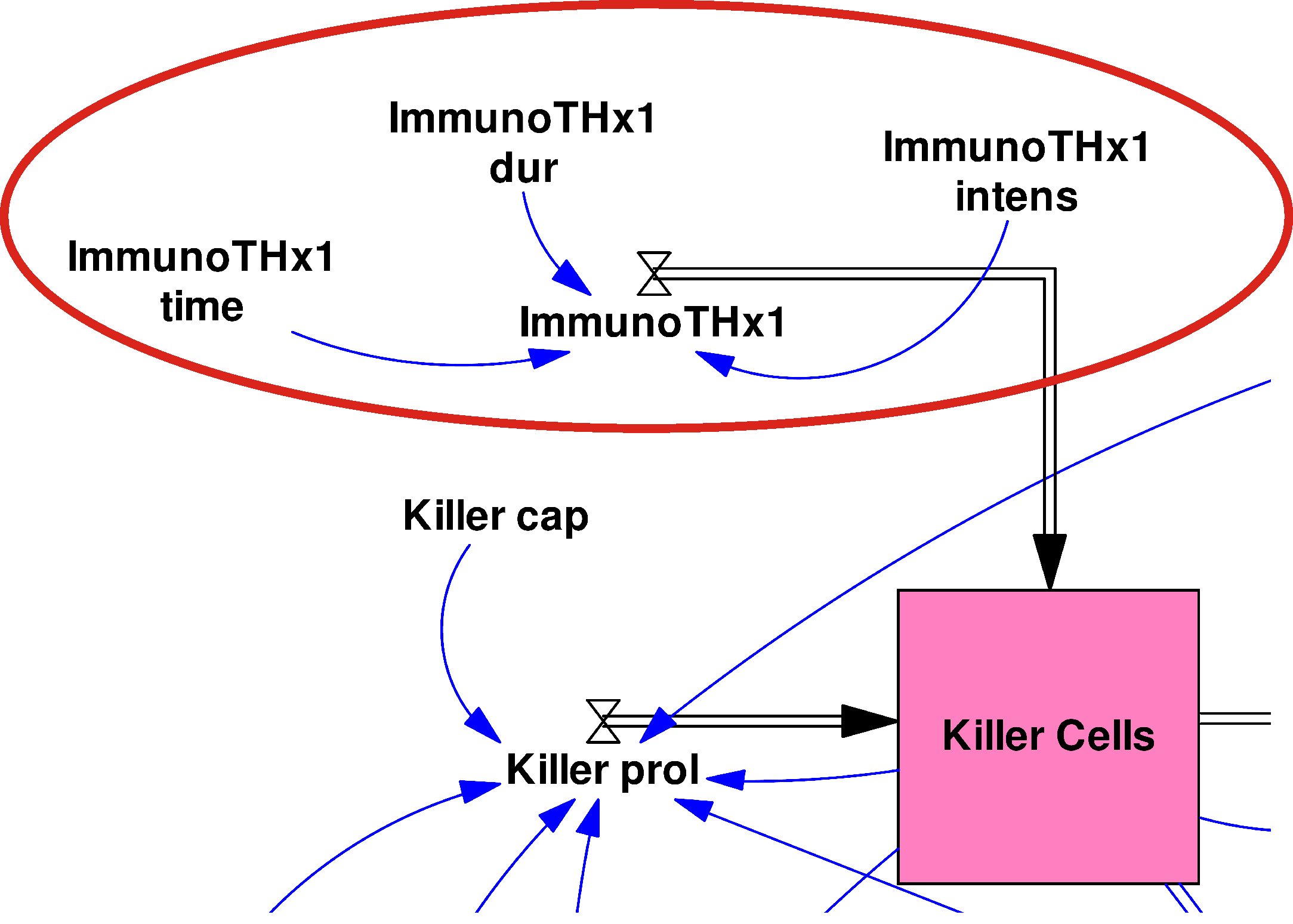

Supplement: S12 Fig — With this extension (adoptive transfer of “Killer Cells”), adoptive immunotherapy (ImmunoTHx1) can be simulated. ImmunoTHx1 increases the number of “Killer Cells” by the factor ImmunoTHx1 intens at time point ImmunoTHx1 time. The parameter ImmunoTHx1 dur can be used for simulation of multiple doses of “Killer Cells.” (TIF) [file pone.0124614.s013.tif]

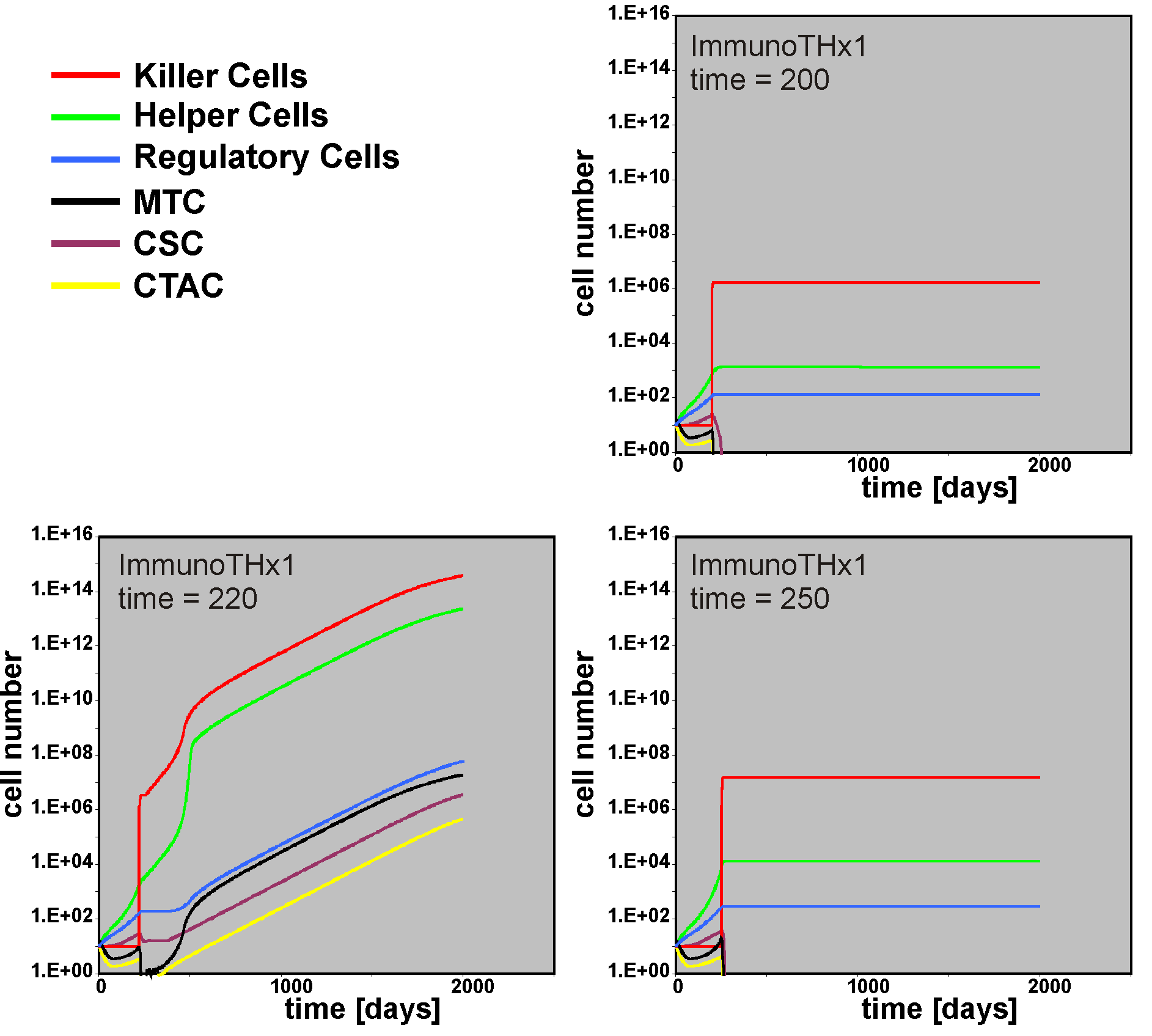

Supplement: S13 Fig — In this simulation, adoptive immunotherapy was simulated by ImmunoTHx intens = 1e+006 and ImmunoTHx dur = 1. Therapy was started at three different time points. (TIF) [file pone.0124614.s014.tif]

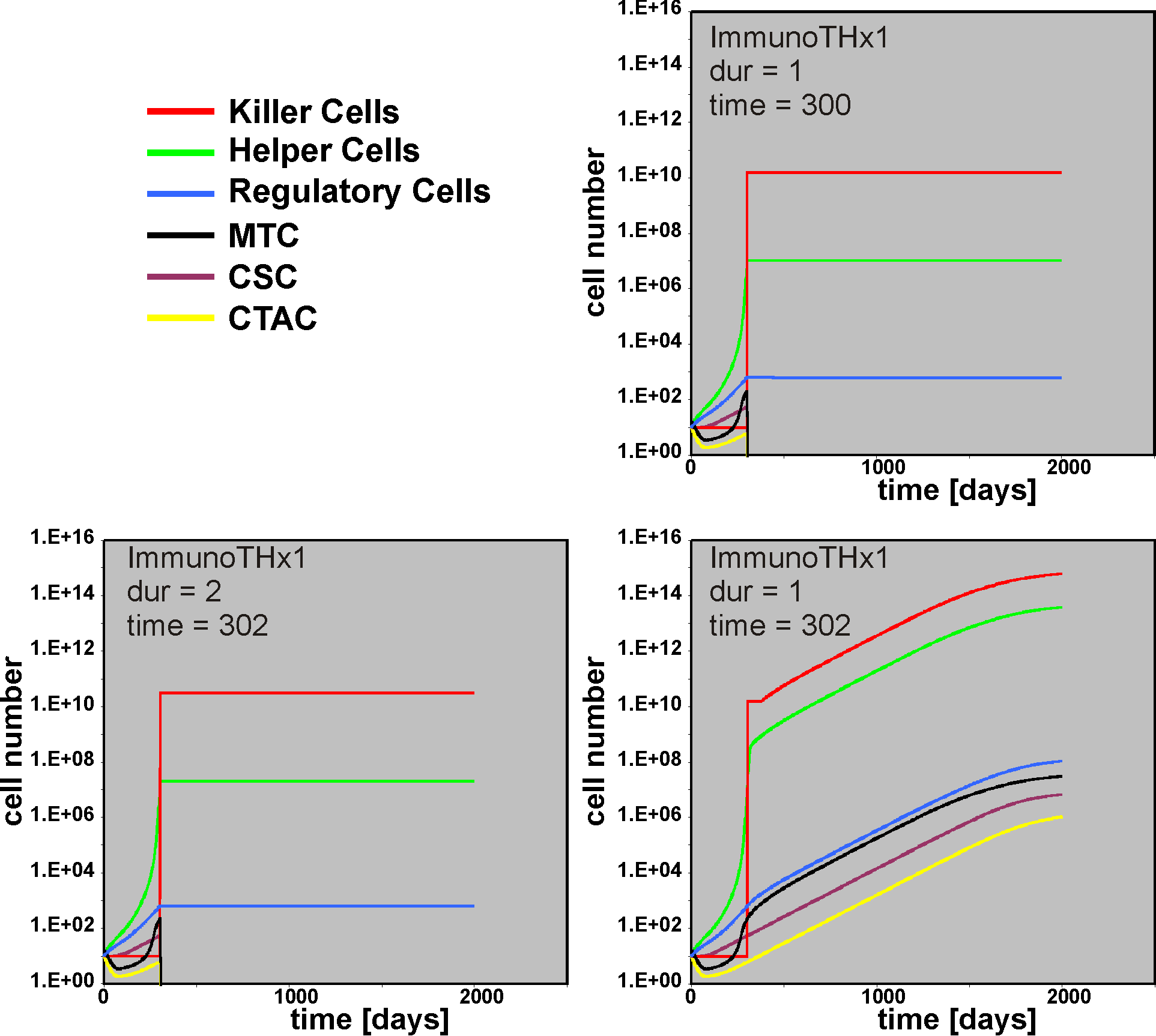

Supplement: S14 Fig — In this simulation, adoptive immunotherapy was simulated by ImmunoTHx1 intens = 1.5e+010. Therapy was started at two different time points. The duration of the therapy was set as 1 or 2 (simulating lower or higher number of single doses of adoptively transferred “Killer Cells”). (TIF) [file pone.0124614.s015.tif]

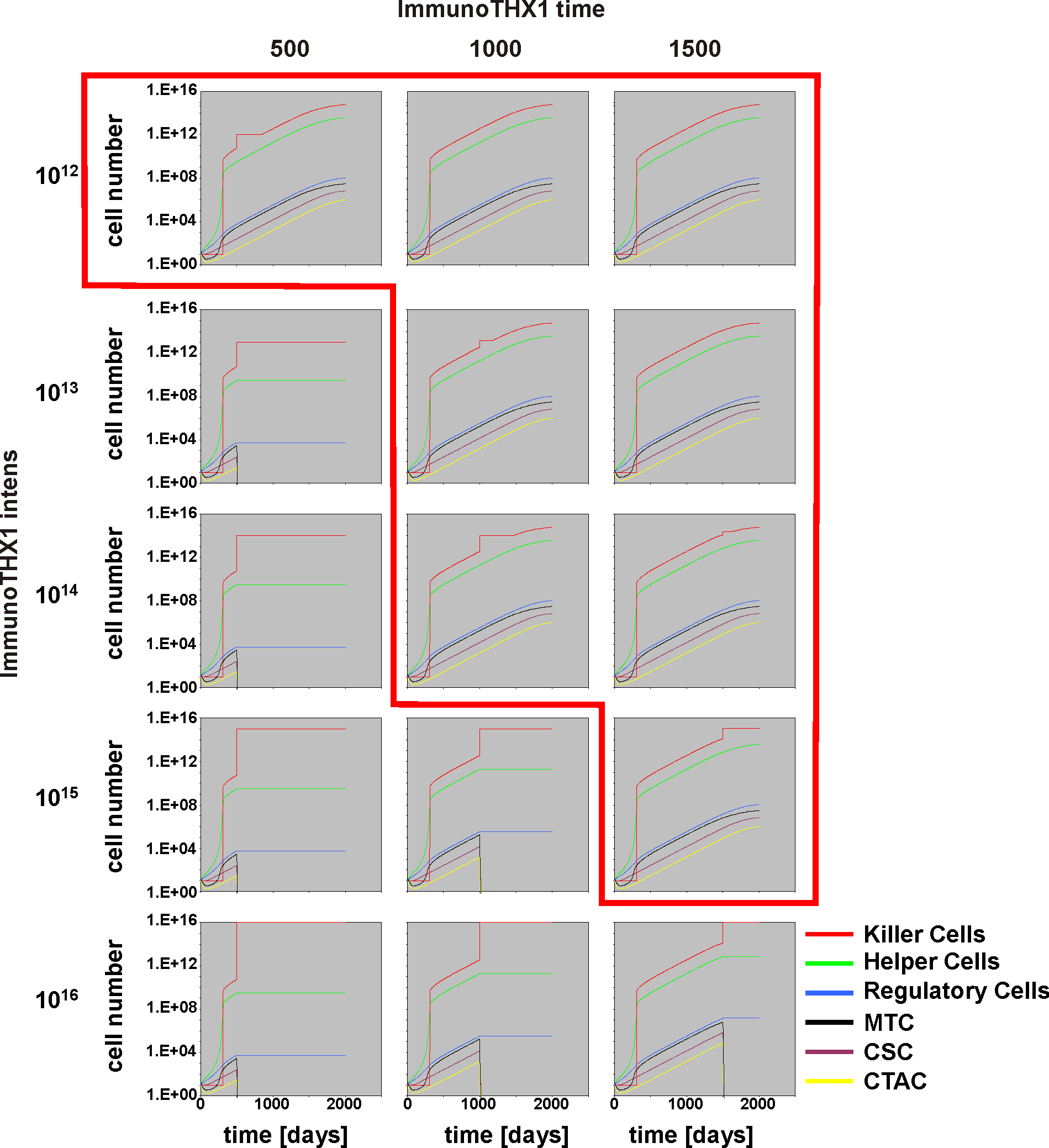

Supplement: S15 Fig — In this simulation, adoptive immunotherapy was simulated by different combinations of ImmunoTHx1 intens (1e+012–1e+016) and ImmunoTHx1 time (500–1,500). The duration of the therapy was set as 1 for all combinations. The red line indicates conditions with tumor growth. (TIF) [file pone.0124614.s016.tif]

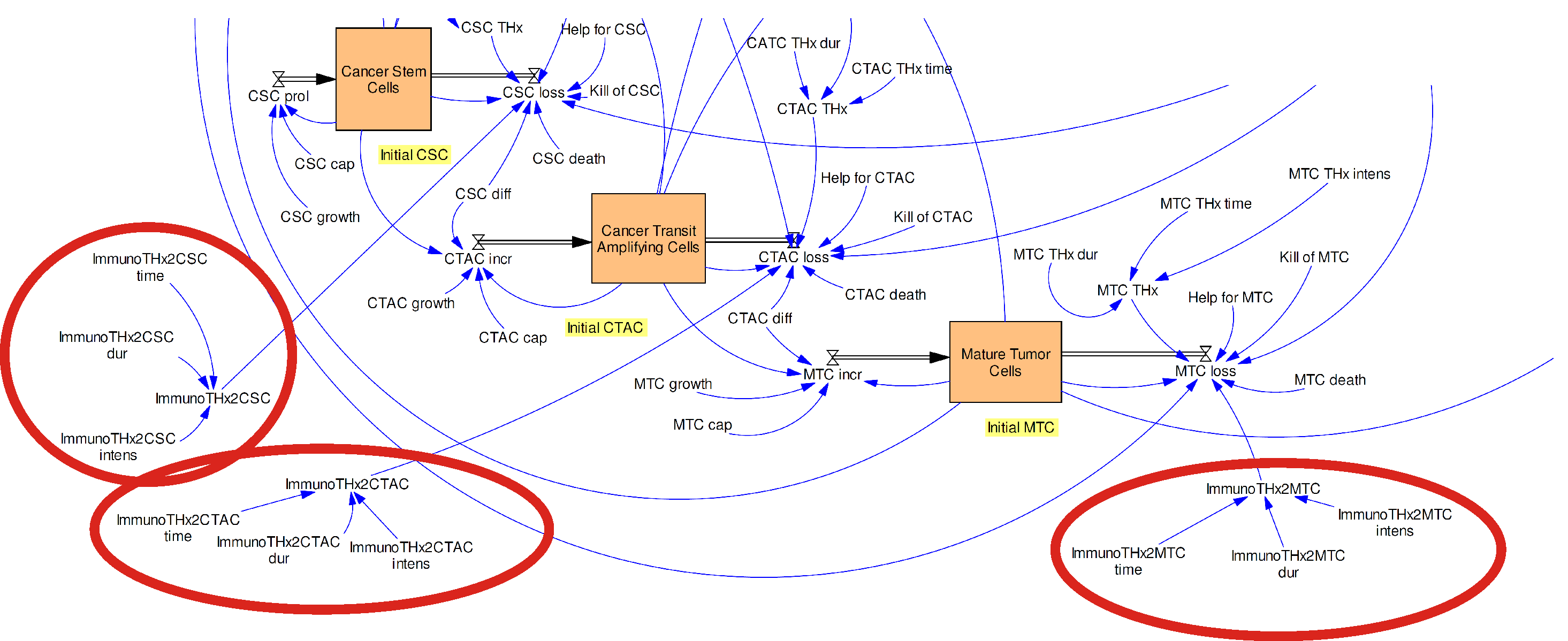

Supplement: S16 Fig — With this extension (increasing the killing activity of “Killer Cells”), the effect of increasing the killing activity of “Killer Cells” (e.g. by vaccination) can be simulated (ImmunoTHx2). ImmunoTHx2 increases the killing capacity of “Killer Cells” for all the types of tumor cells independently. In addition, the time point for ImmunoThx2 as well as the duration of the effect can be set individually for all tumor cell types. With the default values for the duration, killing activity remains stable for 3000 days of the simulation. (TIF) [file pone.0124614.s017.tif]

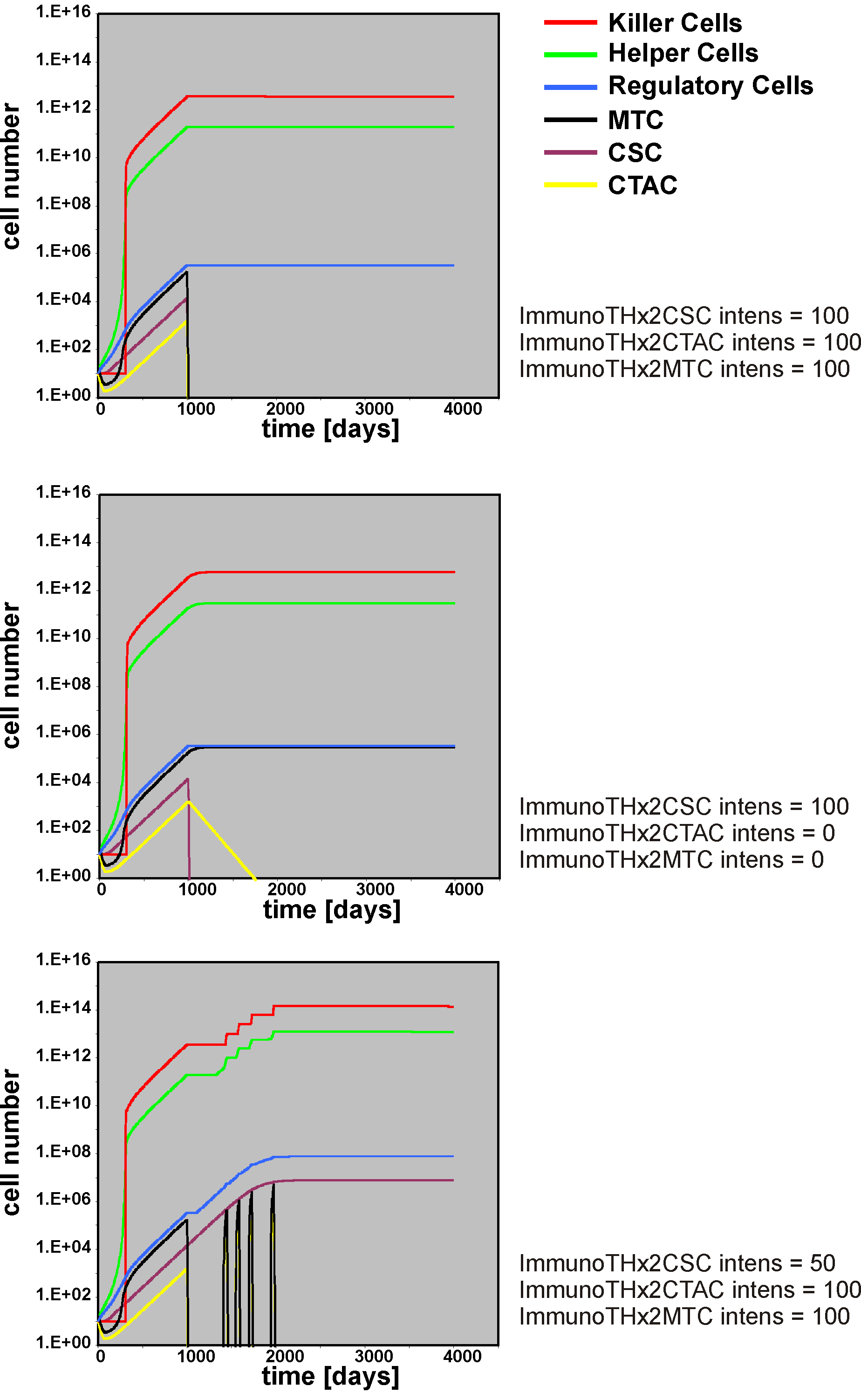

Supplement: S17 Fig — In these simulations, different combinations of ImmunoTHx2CSC intens, ImmunoTHx2CTAC intens and ImmunoTHx2MTC intens were used. Tumor growth stops only after sufficient increase of the killing activity against CSC. The time scale for all simulations in this figure is 4000 days. (TIF) [file pone.0124614.s018.tif]

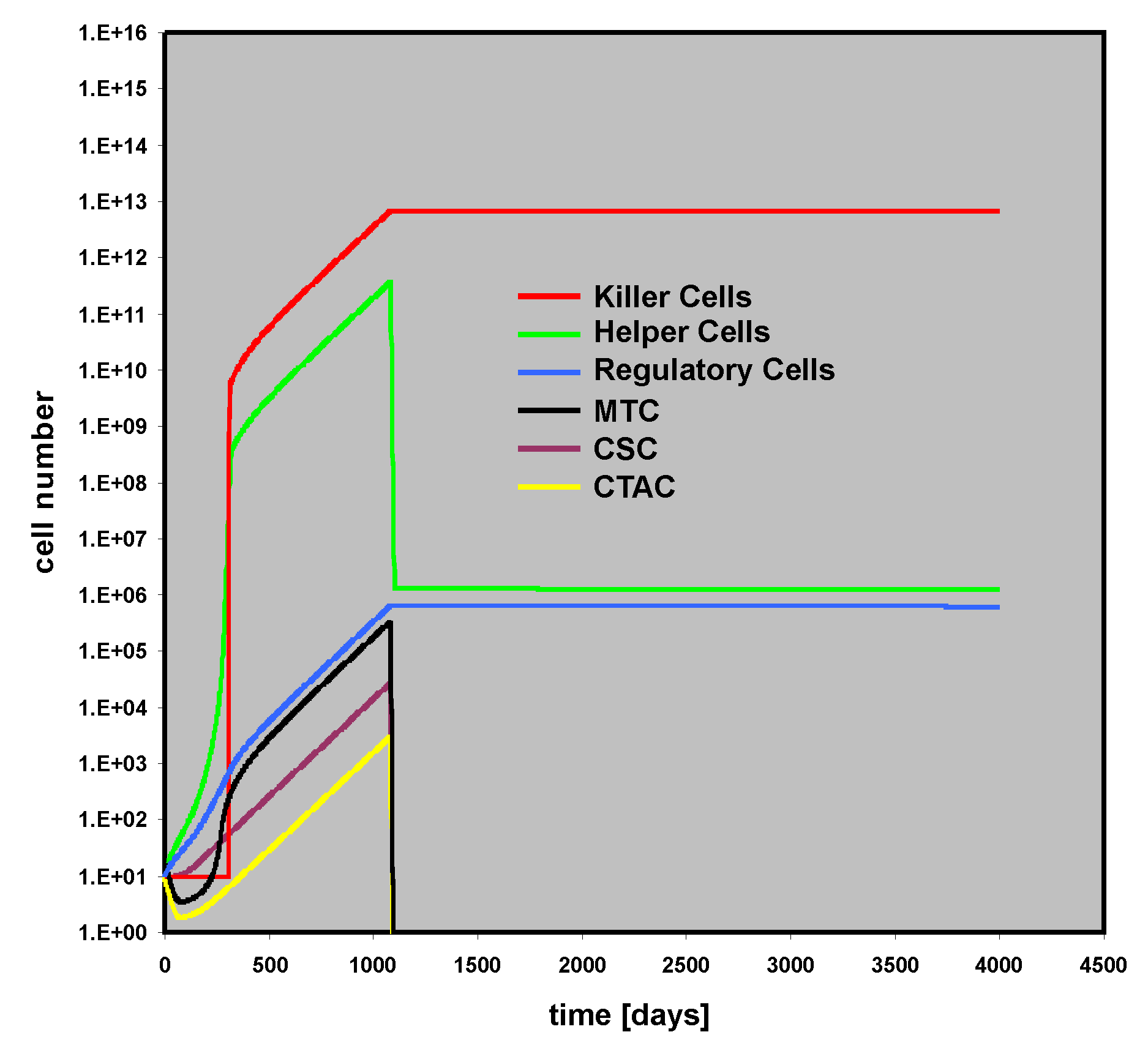

Supplement: S18 Fig — In these simulations, immunotherapy was simulated with ImmunoTHx2CSC intens = ImmunoTHx2CTAC = ImmunoTHx2MTC = 53. This sub-optimal therapy (see also S19 Fig) can be rendered successful by decreasing the number of “Helper Cells” by a “Helper Cell”-specific therapy (Helper THx time = 1000; Helper THX intens = 0.7). The time scale for this simulation is 4000 days. (TIF) [file pone.0124614.s019.tif]

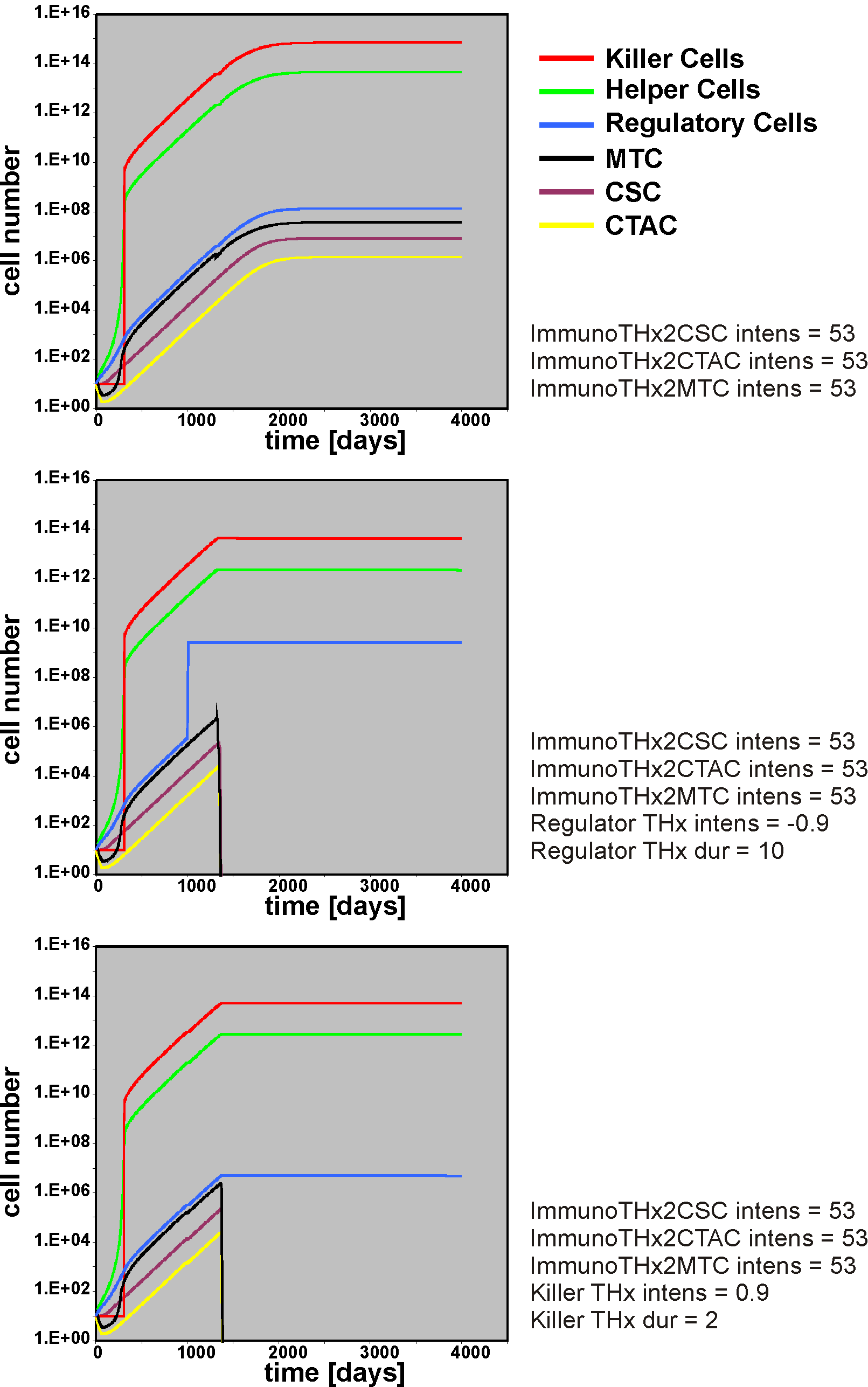

Supplement: S19 Fig — In these simulations, immunotherapy was simulated with ImmunoTHx2CSC intens = ImmunoTHx2CTAC intens = ImmunoTHx2MTC intens = 53. This sub-optimal therapy (upper panel) can be rendered successful by a short pulse of toxic therapy for “Killer Cells” (Killer THx time = 1000; lower panel) or an increase of the number of “Regulatory Cells” (Regulator THx time = 1000; middle panel). The time scale for all simulations in this figure is 4000 days. (TIF) [file pone.0124614.s020.tif]
